# Influence of ND10 Components on Epigenetic Determinants of Early KSHV Latency Establishment

**DOI:** 10.1371/journal.ppat.1004274

**Published:** 2014-07-17

**Authors:** Thomas Günther, Sabrina Schreiner, Thomas Dobner, Uwe Tessmer, Adam Grundhoff

**Affiliations:** 1 Research Group Virus Genomics, Heinrich Pette Institute, Leibniz Institute for Experimental Virology, Hamburg, Germany; 2 Research Unit Viral Transformation, Heinrich Pette Institute, Leibniz Institute for Experimental Virology, Hamburg, Germany; Rosalind Franklin University of Medicine and Science, United States of America

## Abstract

We have previously demonstrated that acquisition of intricate patterns of activating (H3K4me3, H3K9/K14ac) and repressive (H3K27me3) histone modifications is a hallmark of KSHV latency establishment. The precise molecular mechanisms that shape the latent histone modification landscape, however, remain unknown. Promyelocytic leukemia nuclear bodies (PML-NB), also called nuclear domain 10 (ND10), have emerged as mediators of innate immune responses that can limit viral gene expression via chromatin based mechanisms. Consequently, although ND10 functions thus far have been almost exclusively investigated in models of productive herpesvirus infection, it has been proposed that they also may contribute to the establishment of viral latency. Here, we report the first systematic study of the role of ND10 during KSHV latency establishment, and link alterations in the subcellular distribution of ND10 components to a temporal analysis of histone modification acquisition and host cell gene expression during the early infection phase. Our study demonstrates that KSHV infection results in a transient interferon response that leads to induction of the ND10 components PML and Sp100, but that repression by ND10 bodies is unlikely to contribute to KSHV latency establishment. Instead, we uncover an unexpected role for soluble Sp100 protein, which is efficiently and permanently relocalized from nucleoplasmic and chromatin-associated fractions into the insoluble matrix. We show that LANA expression is sufficient to induce Sp100 relocalization, likely via mediating SUMOylation of Sp100. Furthermore, we demonstrate that depletion of soluble Sp100 occurs precisely when repressive H3K27me3 marks first accumulate on viral genomes, and that knock-down of Sp100 (but not PML or Daxx) facilitates H3K27me3 acquisition. Collectively, our data support a model in which non-ND10 resident Sp100 acts as a negative regulator of polycomb repressive complex-2 (PRC2) recruitment, and suggest that KSHV may actively escape ND10 silencing mechanisms to promote establishment of latent chromatin.

## Author Summary

KSHV is the etiological agent of several cancers including Kaposi's sarcoma, one of the most frequent tumors in Sub-Saharan Africa. Since the proliferating cells in these cancers are latently infected with KSHV, there is an urgent need to elucidate the molecular basis underlying latency establishment. While it is well established that intricate histone modification patterns preserve the latent state, the mechanisms that lead to primary establishment of such patterns and subsequent formation of repressive heterochromatin remain largely unknown. During the last years, components of distinct nuclear compartments, so called ND10 or PML bodies, have emerged as modulators of viral chromatin and gene expression. Here, we present the first systematic analysis of the mutual influence between KSHV and ND10 components during the early infection phase. We find that latent KSHV infection dramatically alters the sub-nuclear distribution of the soluble form of a ND10 core protein termed Sp100. This relocalization likely serves to facilitate the recruitment of polycomb repressive complexes and formation of facultative heterochromatin, and we hence propose that soluble Sp100 is an antagonist of KSHV latency establishment. Our findings have important implications for the understanding of viral latency establishment and also provide valuable insight into cellular chromatin regulation pathways.

## Introduction

KSHV, a member of the gammaherpesvirus subfamily, is considered the etiological agent of Kaposi sarcoma (KS) and is furthermore associated with Primary effusion lymphoma (PEL) and a subset of cases of multicentric Castleman's disease (MCD) [1]–[3]. The majority of proliferating tumor cells in these malignancies are latently infected with KSHV and do not produce viral progeny. In such cells, the virus persists as an extrachromosomal episome that undergoes licensed DNA replication and faithful segregation to daughter cells upon cell division. A key mediator of these processes is the latency associated nuclear antigen (LANA), a multifunctional protein which recruits the cellular DNA replication machinery to the latent origin of replication at the onset of S-phase, and which furthermore tethers viral episomes to host chromosomes during meiosis [4]–[13]. While constitutive LANA expression is a principal requirement for establishment and maintenance of latency, expression of lytic genes must be suppressed in order to prevent productive cycle entry. We have previously shown that latent viral chromatin of PEL-derived cell lines as well as *de novo* infected cells exhibits highly distinct patterns of activating and repressive histone modifications [14]. Especially in *de novo* infected cells, the latter comprise almost exclusively tri-methylation of histone H3 at lysine 27 (H3K27me3), a facultative heterochromatin mark that is abundantly present on viral genomes at day 5 of a *de novo* infection. In contrast, DNA methylation patterns require weeks to evolve, indicating that this epigenetic mark does not contribute to primary latency establishment [14]. While the LANA promoter stays exempt from H3K27me3 modification, we and others have observed that the simultaneous presence of activating H3K4me3 and repressive H3K27me3 marks creates a bivalent state of repression at the promoter of the lytic master switch transactivator Rta encoded by ORF50 [14]–[17], suggesting that H3K27me3-mediated suppression of lytic gene transcription is an important feature of viral latency.

*De novo* acquisition of H3K27me3 marks is catalyzed by the methyl-transferase enhancer of zeste homolog 2 (Ezh2), which together with Suz12 and Eed forms the core components of the polycomb repressive complex 2 (PRC2). It is thought that the primary mediator of transcriptional repression at H3K27me3-marked loci is the polycomb repressive complex 1 (PRC1), a multiprotein complex which binds to H3K27me3 via its CBX subunit and subsequently catalyzes ubiquitination of histone H2A at lysine 119 (H2AK119ub). In accord with this model, a recent study found that both PRC1 and H2AK119ub are recruited to H3K27me3-modified regions on the KSHV genome during latency, and that shRNA-mediated depletion of Ezh2 significantly reduces levels of H3K27me3 as well as H2AK119ub and PRC1 occupancy [18]. Although there is considerable knowledge regarding the factors that preserve H3K27me3 when cells divide [19]–[22], the mechanisms that mediate primary PRC2 recruitment in mammalian cells remain poorly understood [20], [23]–[31]. KSHV genomes enter the nucleus in form of epigenetically naïve DNA [14], [32] and thus must newly establish latent chromatin patterns upon each infection cycle. Hence, KSHV latency establishment represents an ideal model to study PRC2 recruitment mechanisms as well as other events that may govern chromatinization of invading DNA molecules.

Nuclear domain 10 (ND10), also called PML nuclear bodies (PML-NB), are discrete nuclear structures involved in a multitude of pathways including protein degradation [33], transcriptional regulation [34], [35], cellular senescence [36]–[39], tumor suppression [40], [41], DNA repair [42], [43], apoptosis [44]–[47] and epigenetic regulation [48]. ND10s are also thought to represent important mediators of innate antiviral defense mechanisms [49]–[51]. This hypothesis is based on the fact that several ND10 associated factors are interferon-stimulated genes [52]–[54], and that ND10s or their components have been found to impair efficient productive replication of a variety of viruses [50], [51], [55]. In accord with this hypothesis, many viruses have developed mechanisms to counteract and overcome repressive properties of ND10s, resulting in elevated levels of viral progeny production [56], [57]. ND10s are nuclear multi-protein complexes with an average size of 0.2 µm–1.0 µm that can be detected in nearly all human cells [58]. According to current models, the PML protein mediates assembly of ND10 by recruiting other constitutive components like Daxx (Death domain associated protein), Sp100 (speckled protein of 100 kDa) or SUMO (small ubiquitin-related modifier) [59]–[61]. Other components may be present under certain conditions [62], and the precise abundance, composition, structure and function of ND10 thus can vary with cellular context [58], [62]–[70]. Posttranslational modification of PML and PML-associated proteins by the small ubiquitin-like protein SUMO plays an integral role in regulating ND10 functions and formation [60], [71], [72]. The higher three-dimensional sphere-like structure of ND10s is formed by interactions between covalently attached SUMO molecules and SIMs (SUMO interaction motifs) that are present on both the PML and Sp100 core components [35], [60], [72] [48], [73]–[75]. SUMO was initially identified as a reversible post-translational modification in the mid 1990s [76]–[81]. SUMO-1 and SUMO-2/3 proteins are conjugated to distinct substrates *in vivo* following cellular stress [82], suggesting differential roles in normal cell metabolism. In humans a fourth gene is coding for SUMO-4, however it is unclear whether its product can be conjugated to other proteins *in vivo* [83]. As for other nuclear structures, protein accumulation at ND10 does not necessarily indicate that these bodies also represent the primary (or only site) of protein activity. For instance, ND10s respond to stress by releasing Daxx and Sp100, leading to an alteration of heat-shock-protein synthesis [84]. Hence, whereas some proteins may be active within ND10 [85], others may initially be sequestered at these structures, leading to the so called depot hypothesis for protein accumulation and function at ND10s [62]. Since viral replication centers are often located near ND10s, however, and since many viruses including KSHV [86]–[89] disrupt or reorganize ND10s during productive replication, it has been proposed that ND10s may serve as mediators of innate immune responses that induce transcriptional silencing of invading nucleic acids. Consequently, it is tempting to speculate that under conditions in which viruses fail to efficiently counteract ND10s immediately after a *de novo* infection, ND10-dependent formation of heterochromatin [48], [90] may promote a state that ultimately leads to the establishment of viral latency. Although proposed for a number of herpesviruses, however, such models have never been rigorously tested in experimental systems. We here present the first such study in which we have performed a detailed investigation of the role of ND10s and ND10 components on the primary establishment of KSHV latency and the acquisition of repressive H3K27me3 marks.

## Results

### The epigenetic landscape of latent KSHV episomes is established independently of virion-associated factors

**Figure 1:**
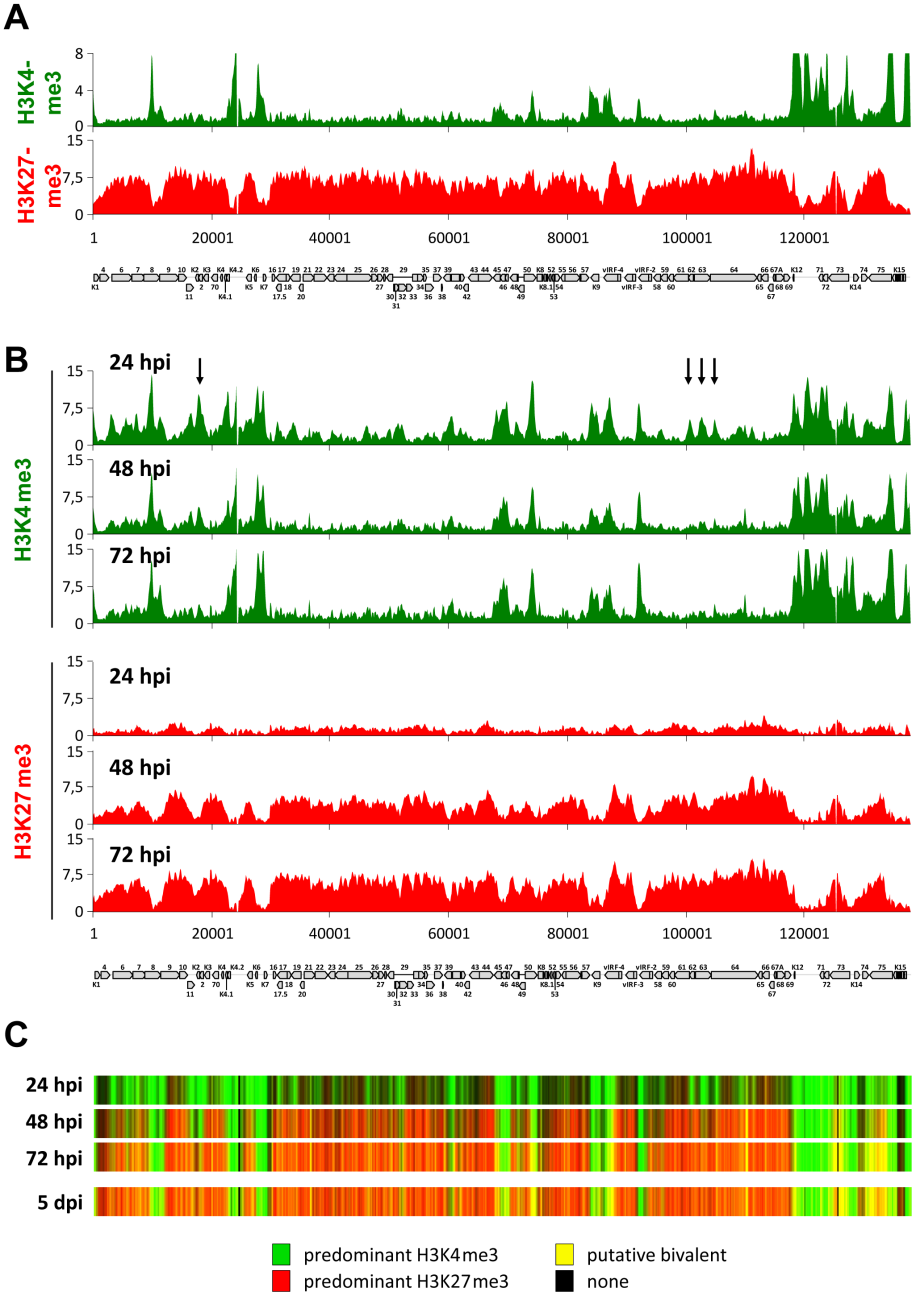
Histone modification profiles acquired by transfected KSHV bacmids and de novo infecting episomes. (**A**) SLK cells were transfected with KSHV BAC16 [143] DNA and selected with hygromycin for 24 days to select for bacmid-carrying cells. Histone modifications were analyzed by high resolution ChIP on microarray analysis with antibodies directed against H3K4me3 (upper panel) or H3K27me3 (lower panel). (**B**) SLK cells were infected with KSHV and chromatin was prepared at indicated time points, and histone modification profiles were investigated by high resolution ChIP on microarray analysis with antibodies directed against H3K4me3 (upper panel) or H3K27me3 (lower panel). Arrows above H3K4me3 profiles denote peaks that are prominent at 24 h post infection, but were diminished upon acquisition of repressive H3K27me3 marks at later time points. Normalized signal intensity values from the profiles shown in A and B as well as from previously investigated SLK cells at 5 d post infection (5 dpi) [14] were used to generate the heat maps shown in (**C**). The heat maps indicate the chromatin status as being either naïve (black), dominated by H3K4me3 (green) or H3K27me3 (red), or characterized by the presence of both modifications (yellow). The latter state is designated as ‘putative bivalent’ since co-occurrence of both modifications on the same nucleosome has formally been proven only for the ORF50/Rta promoter [14].

In order to begin elucidating the mechanisms that control the establishment of latent histone modification patterns, we first sought to clarify whether such patterns are shaped by virion-associated factors and/or signaling events induced by virion binding or entry. For this purpose, we analyzed histone modification patterns acquired by KSHV bacmid DNA (BAC16, kindly provided by J. Jung) after transfection into SLK cells, and compared them to the profiles observed in an authentic infection. We chose SLK cells since we and others have previously shown that this cell line can be efficiently infected with KSHV *in vitro*, resulting in the establishment of tightly controlled latency in the great majority of cells [14], [18], [91]–[93]. Furthermore, we have demonstrated that histone modification and DNA methylation profiles acquired by KSHV genomes in *in vitro* infected SLK cells are near identical to those observed in PEL cells [14], making them an ideal tool to study epigenetic aspects of latency establishment. In Figure 1A, we present high-resolution ChIP-on-chip microarray analyses of H3K4me3 and H3K27me3 patterns across the KSHV genome in BAC16-transfected SLK cells selected with hygromycin for 24 days (due to low initial transfection efficiencies of the bacmid DNA, we were unable to analyze these cultures without prior selection). Indeed, both histone modification profiles were highly similar to those observed in our previous analysis [14] of SLK cells infected for 5 days (Pearson correlation coefficients of 0,85 and 0,87 for H3K4me3 or H3K27me3, respectively), indicating that virion-mediated delivery of KSHV genomes is not a principal requirement for the establishment of latent histone modification patterns.

### Acquisition of activating and repressive histone marks during early latency establishment are temporally separable events

We next sought to investigate the temporal and spatial dynamics of histone mark establishment prior to the previously investigated 5 d time point of a *de novo* infection. In particular, we aimed to i) investigate whether activating and repressive modifications are acquired simultaneously or in a timely ordered fashion, ii) elucidate whether early patterns exhibit plasticity with regard to spatial deposition of modifications and iii) explore whether the KSHV genome may harbor discrete sequence elements that could function as nucleation sites for the acquisition and subsequent spreading of the polycomb-associated H3K27me3 mark, as has been suggested for cellular loci.

To address these points, we performed ChIP-on-chip analyses at several time points during the early phase of a *de novo* KSHV infection in SLK cells. As shown in the upper panels of Figure 1B, we find that the earliest detectable epigenetic event is the emergence of the distinct peak profiles of activating H3K4me3 marks. These profiles were almost fully established at the 24 h time point, after which only a few loci exhibited relative changes in their modification status. The latter exclusively comprise peaks that are present at the earliest time point, but are diminished or eradicated over the following two days (see, for example, the arrow-marked peaks upstream of K2, or the three peaks located between ORFs 61 and 64 in Figure 1B); we did not observe any major peaks that were newly established after the initial 24 h sampling point. In contrast to the activating H3K4me3 modification, H3K27me3 profiles were established with markedly different dynamics, evolving more slowly and not reaching their full extent until 72 h post infection (lower panels in Figure 1B). The overall validity of this finding was furthermore confirmed by conventional ChIP-qPCR performed at a number of selected loci (Figure S1). Interestingly, we did not find any evidence for the hypothesis that the H3K27me3 modification may spread from initial nucleation sites. Instead, all regions that ultimately become H3K27me3-modified acquire the modification simultaneously and in a gradual fashion. As a consequence, the KSHV genome evolves from a predominantly open chromatin state to one that is progressively defined by heterochromatic and putative bivalent chromatin regions (Figure 1C). The majority of changes occur between the 24 h and 48 h post infection; comparison to the chromatin state of the previously investigated [14] SLK cells at 5 days post infection (lower panel in Figure 1C) indicates that establishment of latent chromatin was complete at 72 h post infection.

Although a significant number of loci may acquire a bivalent chromatin state (which we and others have previously confirmed for the Rta promoter), we found H3K4me3 and H3K27me3 patterns to be anti-correlated at all time points (Pearson correlation coefficients of −0.28, −0.45 and −0.57 at 24, 48 and 72 h post infection, respectively). This observation suggests that the few loci which ultimately stay exempt from or acquire only low levels of H3K27me3 are pre-defined by a number of regions that are already occupied by high levels of H3K4me3 early in the infection. Hence, establishment of latent histone modification patterns represents the result of two independent processes: First, local acquisition of distinct activation-associated H3K4me3 peaks, likely due to the binding of sequence specific factors, and second, genome-wide acquisition of repressive H3K27me3 marks, with a preference for regions that do not already carry high levels of H3K4me3. The finding that H3K27me3 marks do not spread, but rather evolve gradually over the entire episome, furthermore indicates that the latter process involves a global mechanism that does not require distinct sequence elements. Given that ND10 have been shown to repress the transcription of many DNA viruses via chromatin-based mechanisms, we considered the possibility that ND10 or individual ND10 components may contribute to the repression of KSHV episomes during early latency establishment.

### KSHV episomes do not reside near ND10 bodies during the early phase of a *de novo* infection

**Figure 2:**
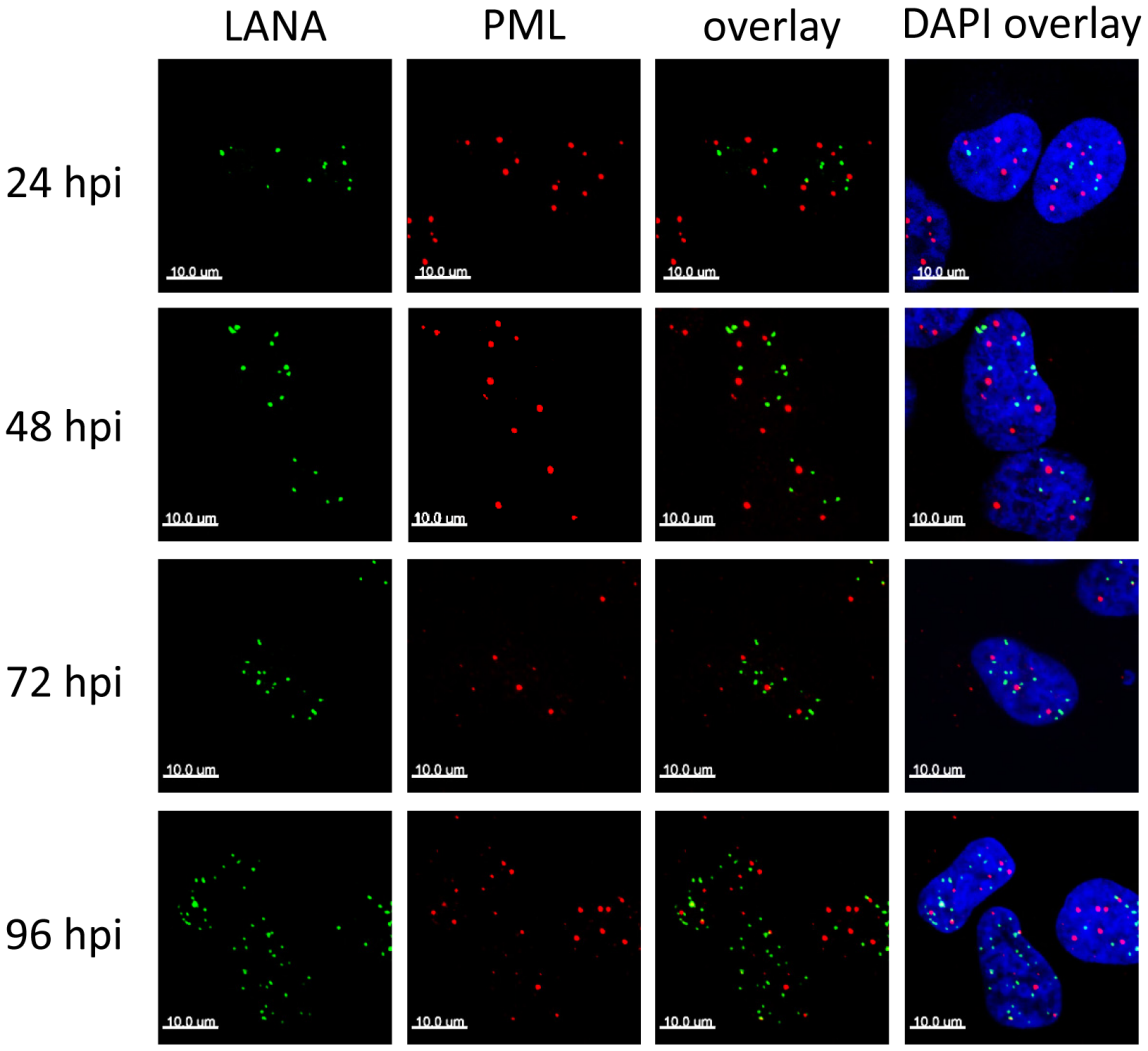
LANA foci do not co-localize with ND10/PML. SLK cells were infected with KSHV, fixed at the indicated time point, and analyzed for LANA and PML using standard IF staining and confocal microscopy procedures. DAPI images represent one confocal plane, whereas LANA and PML are depicted as maximum intensity projections to demonstrate separate localization of both proteins in all three dimensions.

In latently infected PEL cells, the LANA protein accumulates in nuclear dot-like structures that perfectly co-localize with viral episomes [10], [94], a finding which we formally confirmed for *de novo* infected SLK cells (Figure S2). Likewise, in accord with previous studies performed in latently infected PEL cells [95], [96], we observed that long-term latently infected SLK cells harbor intact PML bodies which do not co-localize with LANA foci (data not shown), suggesting that ND10s do not directly contribute to the repression of lytic genes during KSHV latency. However, it seemed possible that KSHV episomes may transiently communicate with ND10 during the establishment phase, after which the viral genomes may be released while maintaining their repressed state in a ND10-independent manner. If so, we would expect to observe co-localization of LANA and ND10 especially around the 24 and 48 h time points, when repressive marks are acquired. To test this hypothesis, we determined the sub-nuclear localization of LANA and the ND10 core component PML during the early stages of a *de novo* infection by confocal immunofluorescence (IF) microscopy. As shown in Figure 2, both proteins were distributed in spatially distinct speckled patterns, with no significant co-localization being detectable at any given time point. Hence, KSHV does not disrupt ND10 in latently infected SLK cells, and the process of *de novo* latency establishment does not involve a transient physical interaction with intact ND10 during the time period when H3K27me3 marks accumulate on KSHV episomes.

### KSHV infection displaces Sp100, but not Daxx or PML from soluble protein fractions

**Figure 3:**
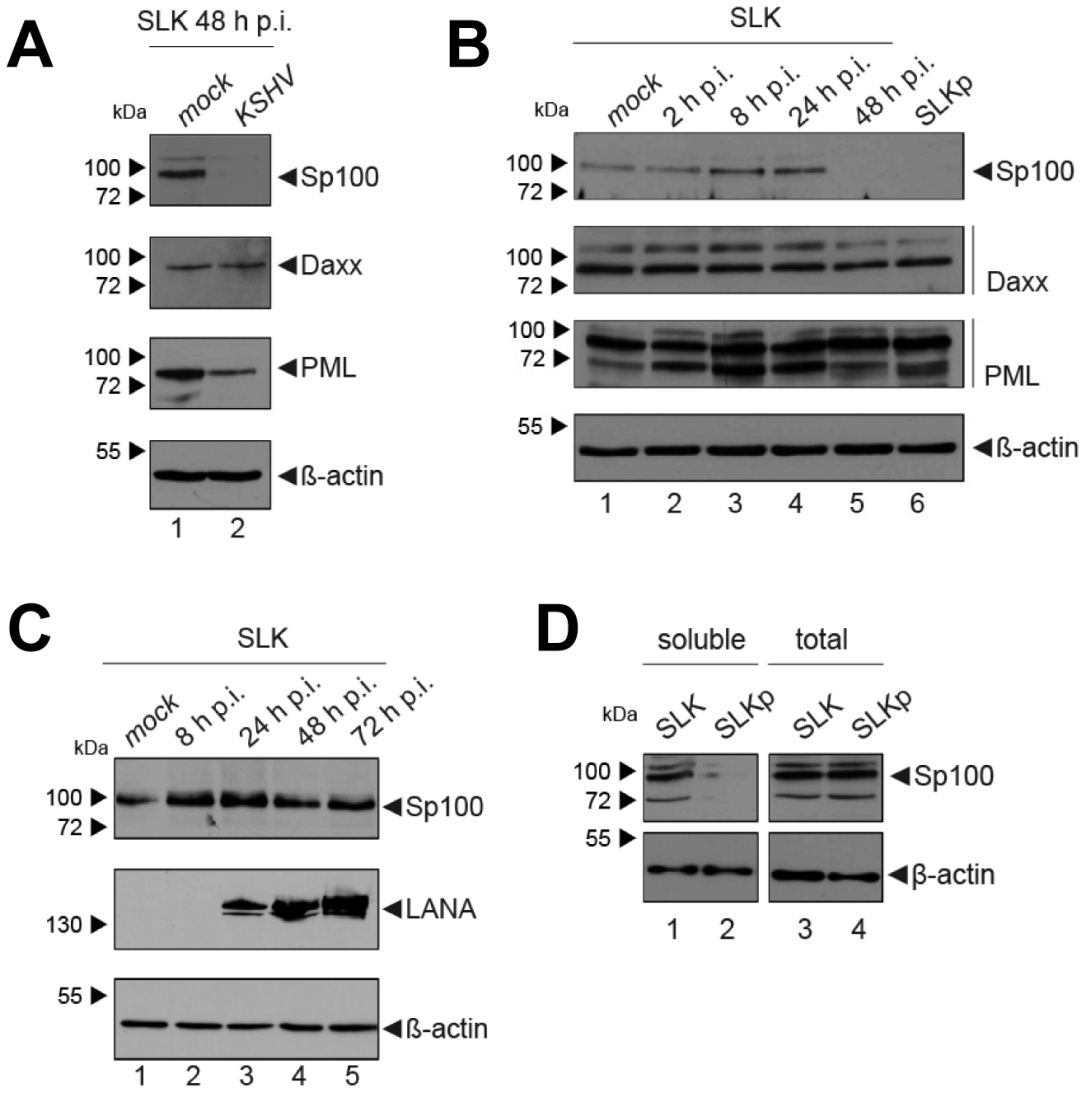
Soluble Sp100 levels are reduced upon KSHV infection of SLK cells. (**A**) Analysis of low-salt soluble RIPA extracts prepared from mock infected or KSHV infected SLK cells after 48 hours. (**B**) Analysis of soluble low-salt RIPA extracts prepared from long-term KSHV infected SLK cells (SLKp, lane 6), mock infected SLK cells (lane 1), or SLK cells that had been infected with KSHV for the indicated time points (lanes 2–5). (**C**) Analysis of total protein extracts prepared from mock infected SLK cells (lane 1) or SLK cells infected with KSHV for the indicated time points. (**D**) Analysis of soluble low-salt extractable (left panel) or total (right panel) protein fractions in uninfected (SLK) or long-term KSHV infected SLK cells (SLKp). β-actin served as a loading control in all panels.

**Figure 4:**
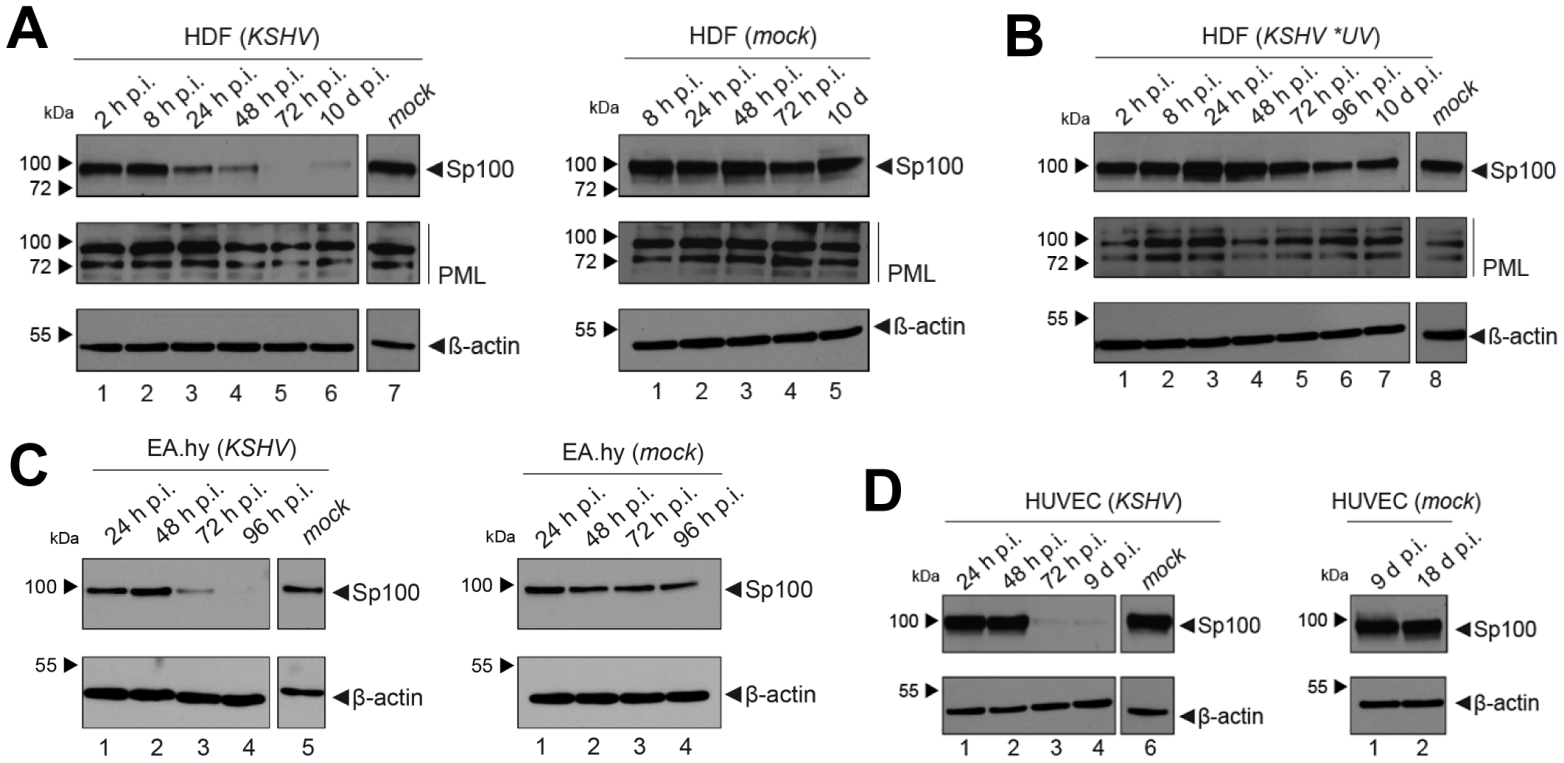
Soluble Sp100 levels are reduced upon KSHV infection of HDF, EA.hy and HUVEC cells. Western blot analysis of low-salt soluble RIPA extracts prepared from: (**A**) HDF cells that had been infected with KSHV for the indicated time points (left panel, lanes 1–6) or mock infected cells at 0 h (left panel, lane 7) or between 8 h and 10 days post treatment (right panel, lanes 1–5), (**B**) mock infected HDF cells (lane 8) or HDF cultures exposed to UV-irradiated KSHV supernatants (lanes 1–7), (**C**) KSHV or mock infected EA.hy cells and (**D**) KSHV or mock infected HUVEC cells. β-actin served as a loading control in all panels.

After having investigated the influence of KSHV latency on ND10, we next aimed to determine whether KSHV infection may influence individual ND10 components on the protein level, preferentially in the protein fraction that can be solubilized under low-salt conditions. We reasoned that such changes may contribute to KSHV latency establishment without resulting in a microscopically detectable alteration of ND10s. We therefore infected SLK cells for 48 hours and prepared low-salt RIPA extracts, followed by detection of the ND10 core proteins PML, Daxx and Sp100 by Western blot (WB) analysis. Surprisingly, we observed that KSHV infection resulted in the complete removal of Sp100 from soluble extracts (Figure 3A, top panel) at 48 h post infection. In contrast, protein levels of PML, Daxx and a control protein (β-actin) remained largely unaffected (see lower panels in Figure 3 A). To further investigate the temporal dynamics of this phenomenon, we performed a time course experiment with samples collected after 2, 8, 24, and 48 h of infection, and additionally analyzed SLKp cells (a pool of long-term infected single cell clones that stably maintains a wt KSHV infection without any selection [91]). The time course experiment confined the disappearance of soluble Sp100 to a time period between 24 and 48 h post infection. (Figure 3B, lanes 2 to 5). As shown in lane 6 of Figure 3B, SLKp cells were furthermore completely devoid of soluble Sp100, indicating that the phenotype is stable in long-term latently infected cells. To investigate whether depletion of Sp100 was specific for soluble fractions, we next analyzed total protein extracts. Indeed, total protein levels in *de novo* (Figure 3C) SLK cells as well long-term infected SLKp cells (right panel in Figure 3D) were not diminished when compared to the mock control or uninfected SLK cells, respectively. Instead, we observed that total Sp100 protein levels exhibited a mild increase at 8 and 24 hours post infection, but had returned to baseline levels at the 48 h time point. Detection of LANA in these fractions furthermore demonstrated that the protein had accumulated to detectable levels at the 24 h time point, i.e. the time after which Sp100 disappears from soluble fractions (center panels in Figure 3C). To exclude the possibility that the observed effects may be specific for SLK cells, we next sought to confirm our results in other cells. For this purpose, we chose EA.hy 926 cells, a somatic hybrid of endothelial cells and a lung carcinoma line, as well as primary human dermal fibroblasts (HDF) and primary human umbilical vein endothelial cells (HUVEC). Similar to SLK cells, EA.hy, HDF and HUVEC cells can be infected with KSHV with high efficiency, resulting in the establishment of latency in the great majority of cells [91]. As for SLK cells, immunofluorescence analysis of all *de novo* infected cell cultures confirmed readily detectable LANA expression in ∼90 to 95% of cells at 48 h post infection (data not shown). Indeed, similar to the observations made in SLK cells, Sp100 became undetectable in low-salt RIPA extracts at 72 (HDF, HUVEC) or 96 h (Ea.hy) post infection in KSHV infected, but not in mock infected cultures (Figure 4A, -C and -D).

### Virion-associated factors, proteasomal degradation or transcriptional silencing are not responsible for loss of soluble Sp100

Since substantial diminishment of soluble Sp100 levels did not set in before approx. 24 to 48 h post infection, and since furthermore the phenotype persisted in long-term infected SLK cells, we deemed it unlikely that signaling events induced by virion binding or entry were responsible for the observed effects. However, given that tegument proteins including, e.g. ORF3 of herpesvirus saimiri (HVS), have been shown to induce proteosomal degradation of Sp100 [97], it seemed formally possible that virion-associated proteins may induce Sp100 depletion in a delayed manner. In order to test this hypothesis, we infected HDF cells with an UV-inactivated virion preparation. To avoid excessive irradiation (which may potentially result in cross-linking and functional inactivation of virion proteins), we carefully titrated the UV-dosage such that *de novo* expression of viral genes was significantly reduced but not completely abrogated, resulting in the appearance of visible LANA dots in approximately 0.1 to 1% of cells (data not shown). Indeed, when HDF cultures were inoculated with such virion preparations, we did not observe a significant decrease of Sp100 levels relative to mock infected cells (Figure 4B). Hence, *de novo* viral gene expression is required for displacement of Sp100 from soluble fractions in *de novo* latently infected cells.

**Figure 5:**
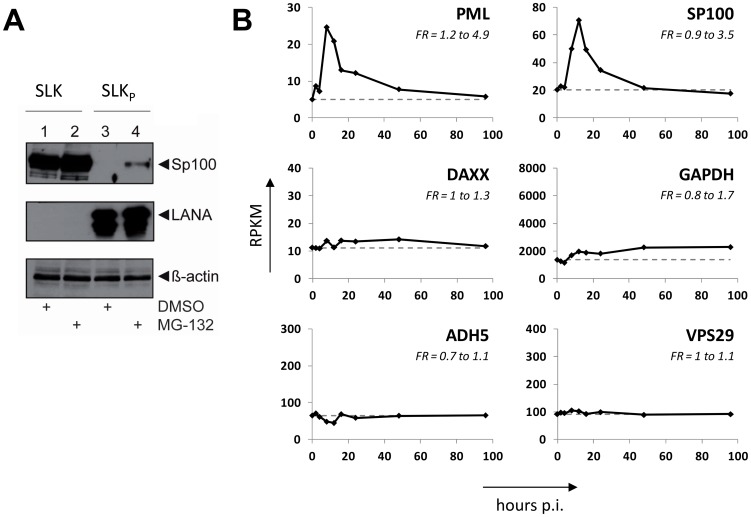
Proteasomal degradation and transcriptional silencing are not responsible for loss of soluble Sp100. (**A**) KSHV negative SLK cells or long-term infected SLK_P_ cells were treated with either the proteasome inhibitor MG-132 or DMSO for 8 h. Subsequently, soluble RIPA extracts were prepared and analyzed for Sp100 and LANA protein levels by immunoblotting. (**B**) Transcript levels of genes encoding the ND10 core components (PML, SP100, DAXX) and the three housekeeping genes GAPDH, ADH5 and VPS29. Transcript levels were analyzed by RNAseq (see complete dataset in Dataset S1) in mock infected (0 h value) or KSHV infected SLK cells after 2, 4, 8, 12, 16, 24, 48 or 96 hours of infection and are given as RPKM (reads per kilobase and million mapped reads) values. Baseline expression levels as observed in mock infected cells are marked across plots by a dashed gray line. The fold range (FR) of maximum up- or down-regulation across the entire time course is indicated in each panel.

Although the fact that KSHV infection did not affect total Sp100 levels argued against the involvement of proteasomal degradation pathways, we considered it formally possible that soluble and insoluble Sp100 fractions may differ in their stability. If so, altered proteasomal degradation pathways may shift the balance towards the insoluble fraction. To investigate this possibility, we treated uninfected SLK cells and long-term infected SLK_P_ cells with the proteasome inhibitor MG-132 for 8 h. As shown in Figure 5A, proteasome inhibition led only to a slight increase of soluble Sp100 protein levels, but failed to restore them to levels comparable to those in uninfected cells (compare lanes 2 and 4 in Figure 5A). We attribute the slight increase in MG-132 treated cells to pleiotropic effects and concluded that proteasomal degradation is unlikely to be responsible for the disappearance of Sp100 from soluble extracts of latently infected cells. We next wondered whether KSHV-induced transcriptional silencing of the Sp100 gene may be responsible for our observations. To investigate this possibility, we inspected levels for these factors in a time course RNA-seq experiment performed at 0, 2, 4, 8, 12, 16, 24, 48 and 96 h post infection (the complete dataset of expressed genes is given in Dataset S1). As shown in Figure 5B, for PML as well as Sp100 (but not Daxx) we observed a transient transcriptional increase, with a marked peak at the 8 h time point. Transcript levels declined thereafter and had returned to approximately baseline by 48 h post infection. Neither transcript was further reduced, clearly indicating that loss of soluble Sp100 was not due to transcriptional repression. Further analysis and functional annotation of all up- and downregulated host genes (Figures S3 and S4 and Datasets S2, S3 and S4) strongly suggest that the transient increase in Sp100 and PML transcription is due to a pronounced but transient interferon response that is largely ablated at approximately 48 h post infection. These results suggest that transient interferon responses are likely responsible for the moderate increase of total Sp100 protein levels observed at the 8 and 24 h time points (see Figure 3C).

### KSHV infection induces massive relocalization of soluble Sp100

**Figure 6:**
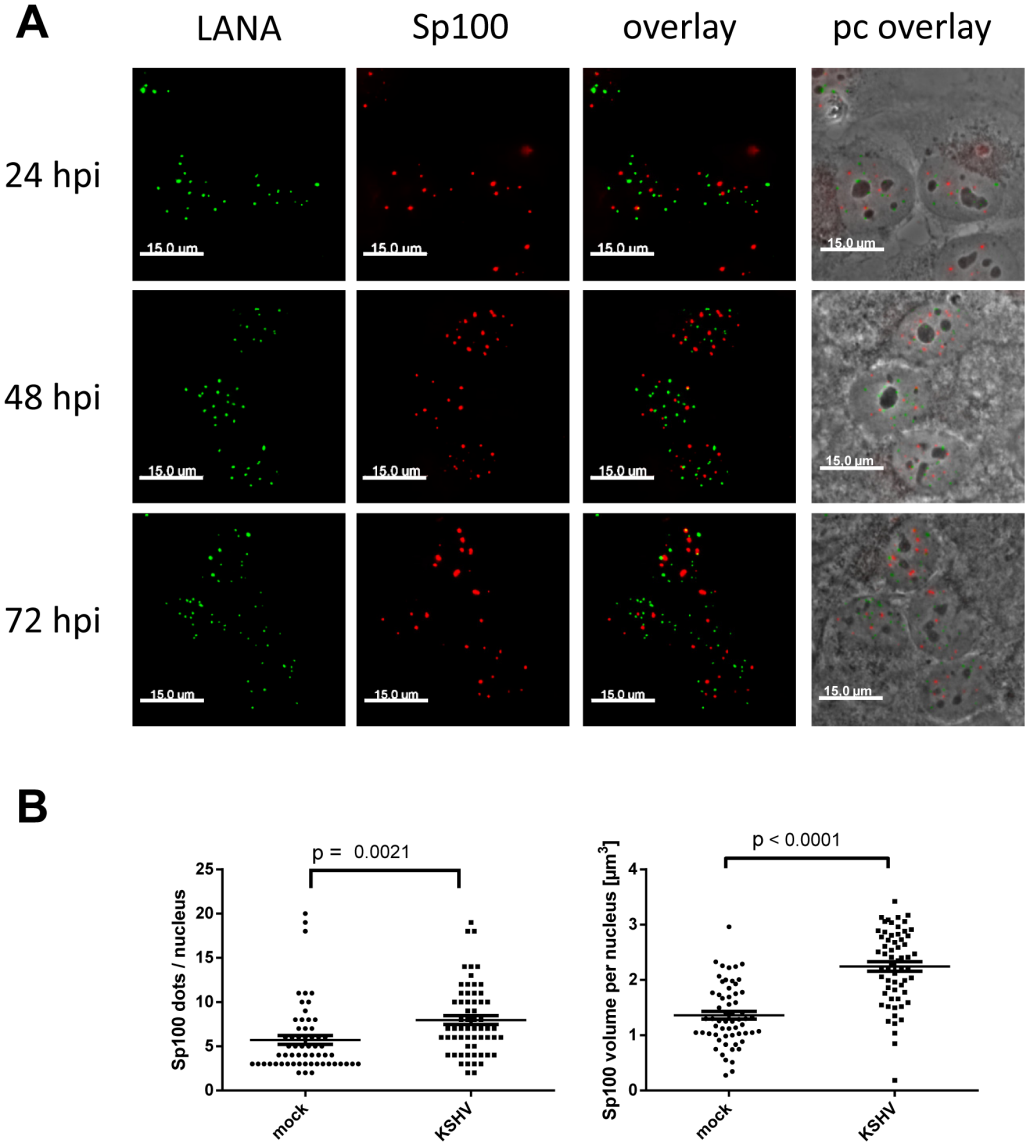
LANA does not co-localize with Sp100, and KSHV infection does not disperse Sp100 from ND10s. (**A**) SLK cells were infected with KSHV and analyzed for LANA and Sp100 using standard IF staining procedures. LANA and Sp100 are depicted as maximum intensity projections from z-stacks to demonstrate separate localization of both proteins in all three dimensions. (**B**) The number of Sp100 containing dots per nucleus (left panel) and total volume of Sp100 dots (sum of volume of all dots within each individual nucleus; right panel) were determined in >60 cells in mock infected or KSHV infected cells at 72 h post infection using the Volocity software (see material and methods for details). Each dot represents a single cell. Bars represent mean and SEM values.

Given the observed reduction of soluble Sp100 - but not total Sp100 or PML - protein levels, we considered it unlikely that Sp100 protein stored in ND10 bodies (which are not efficiently solubilized by low salt RIPA-based extraction) was depleted by KSHV infection. However, to also formally exclude this possibility, we performed IF analysis for Sp100 and LANA at different time points after *de novo* infection of SLK cells. As shown in Figure 6A, Sp100 exhibited typical speckled patterns in mock as well as KSHV-infected cells. Co-staining with PML showed a complete overlap, confirming that microscopically visible Sp100 speckles correspond to ND10 bodies (Figure S4). Similar to PML (Figure 2), we found that Sp100 foci did not co-localize with LANA dots. Likewise, we did not detect a significant reduction in number or size of Sp100-positive foci; instead, we found both number as well as total volume of Sp100 foci to be moderately (but significantly) increased 72 h post infection (Figure 6B). Hence, latent KSHV infection does neither result in global Sp100 dispersal from ND10 bodies, nor in the appearance of a Sp100-depleted ND10 sub-population. These findings are in accord with a previous study that observed eviction of Sp100 in lytically reactivated, but not in latently infected iSLK cells [89].

**Figure 7:**
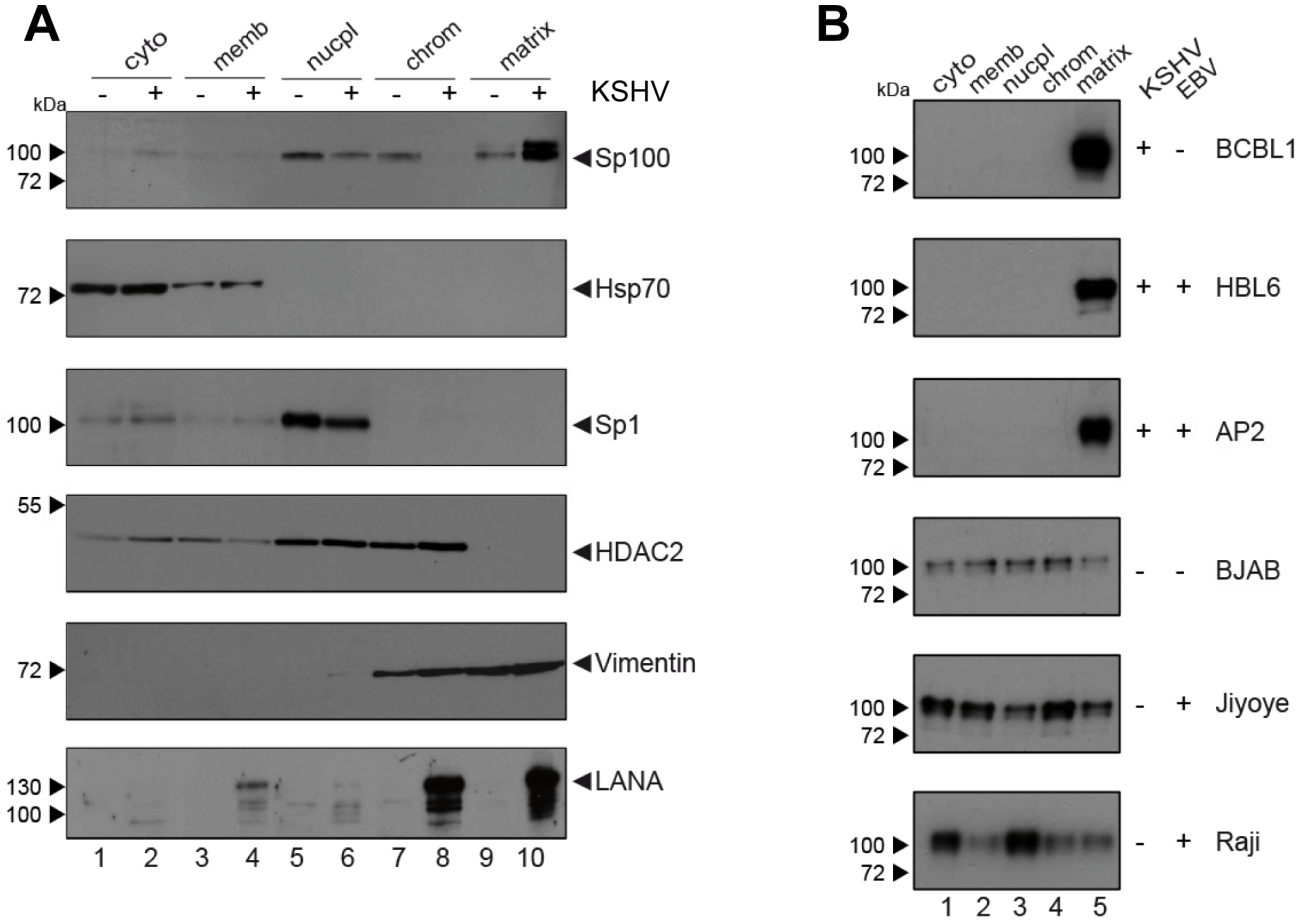
Sp100 accumulates in the insoluble matrix upon *de novo* infection and in PEL-derived B-cell lines. Western-blot analysis of sub-fractionated cellular extracts (*cyto*: cytoplasmic; *memb*: membrane-associated; *nucpl*: nucleoplasmic; *chrom*: chromatin-associated; *matrix*: associated with the insoluble matrix) from: (**A**) EA.hy cells *de novo* infected with KSHV and harvested 72 h after infection or (**B**) PEL-derived (BCBL1, HBL6, AP2) or BL-derived (BJAB, Jijoye, Raji) B cell lines. For each B cell line, infection status for KSHV and EBV is indicated to the right of the panel. Successful fractionation was confirmed by detection of specific marker proteins (Hsp70, Sp1, HDAC2 and Vimentin).

Since i) soluble, but not ND10-bound Sp100 was depleted in KSHV infected cells and depletion was not mediated by ii) proteosomal degradation or iii) transcriptional changes, we reasoned that the observed phenotype most likely resulted from selective relocalization of the low-salt extractable fraction of Sp100 protein. To further investigate this hypothesis, we performed subcellular fractionation of *de novo* infected EA.hy cells at 72 h post infection and subjected the extracts to western blot analysis with antibodies directed against Sp100, LANA and several marker proteins. In accord with previous observations [98], LANA was found in chromatin-bound as well as insoluble nuclear matrix fractions (bottom panel in Figure 7A). As expected, the marker proteins Hsp70 and Sp1 or HDAC2 (center panels in Figure 7A) were found in cytoplasmic and nuclear fractions, respectively; their subcellular distribution was furthermore not affected by KSHV infection (note that, since equal protein amounts from each fraction were analyzed, relative signal levels cannot be readily compared between fractions but only between mock and KSHV-infected samples within each fraction). In contrast, while Sp100 could be readily detected in nucleoplasmic, chromatin-bound and matrix-associated fractions in mock infected cells, we observed a dramatic increase of matrix-associated Sp100 in KSHV infected cells, concomitant with a complete loss of the protein from the chromatin fraction, as well as a slight (but reproducible) decrease in the nucleoplasmic fraction. Hence, KSHV infection induces massive relocalization of low-salt extractable (likely non-ND10 resident) nuclear Sp100 fractions from chromatin-bound fractions (as well as partial relocalization of nucleoplasmic fractions) into the insoluble matrix. We confirmed this phenotype in *de novo* infected SLK cells (data not shown), suggesting that relocalization of Sp100 was responsible for the disappearance of soluble Sp100 in all investigated cell lines and primary cells.

The fact that soluble Sp100 was diminished in long term latently infected SLK_P_ cells (Figure 3B and -D) suggested that relocalization persists throughout latency. To further substantiate this hypothesis in a biologically more relevant system, we performed fractionated western blot analysis of three KSHV-positive PEL-derived cell lines (BCBL1, HBL6 and Cro-AP/2) as well as three KSHV-negative Burkitt Lymphoma (BL) cell lines (BJAB, Jijoye and Raji). Indeed, as shown in Figure 7B, in all three PEL lines Sp100 was almost exclusively detected in the insoluble matrix fraction, irrespective of whether these lines were positive for KSHV only (BCBL1) or co-infected with KSHV and EBV (HBL6 and Cro-AP/2). In contrast, Sp100 was detected in all subcellular fractions prepared from EBV-negative (BJAB) as well as EBV-positive (Jijoye, Raji) BL-derived cell lines (lower panels in Figure 7B). Hence, persistent relocalization of Sp100 into the insoluble matrix represents a KSHV specific phenomenon that is not shared by the closely related gamma-herpesvirus EBV. It is noteworthy that the phenotype in long term infected PEL derived cells was even more dramatic than in *de novo* infected EA.hy cells, which, at least in the experiment shown in Figure 7A, still contained detectable levels of nucleoplasmic Sp100 at 72 h post infection. This may be due to different cellular backgrounds that facilitate relocalization e.g. in B cells; alternatively, it is possible that the balance may progressively shift towards the matrix in a time dependent fashion.

### LANA is sufficient to induce Sp100 SUMOylation and to relocalize Sp100 into the insoluble matrix

**Figure 8:**
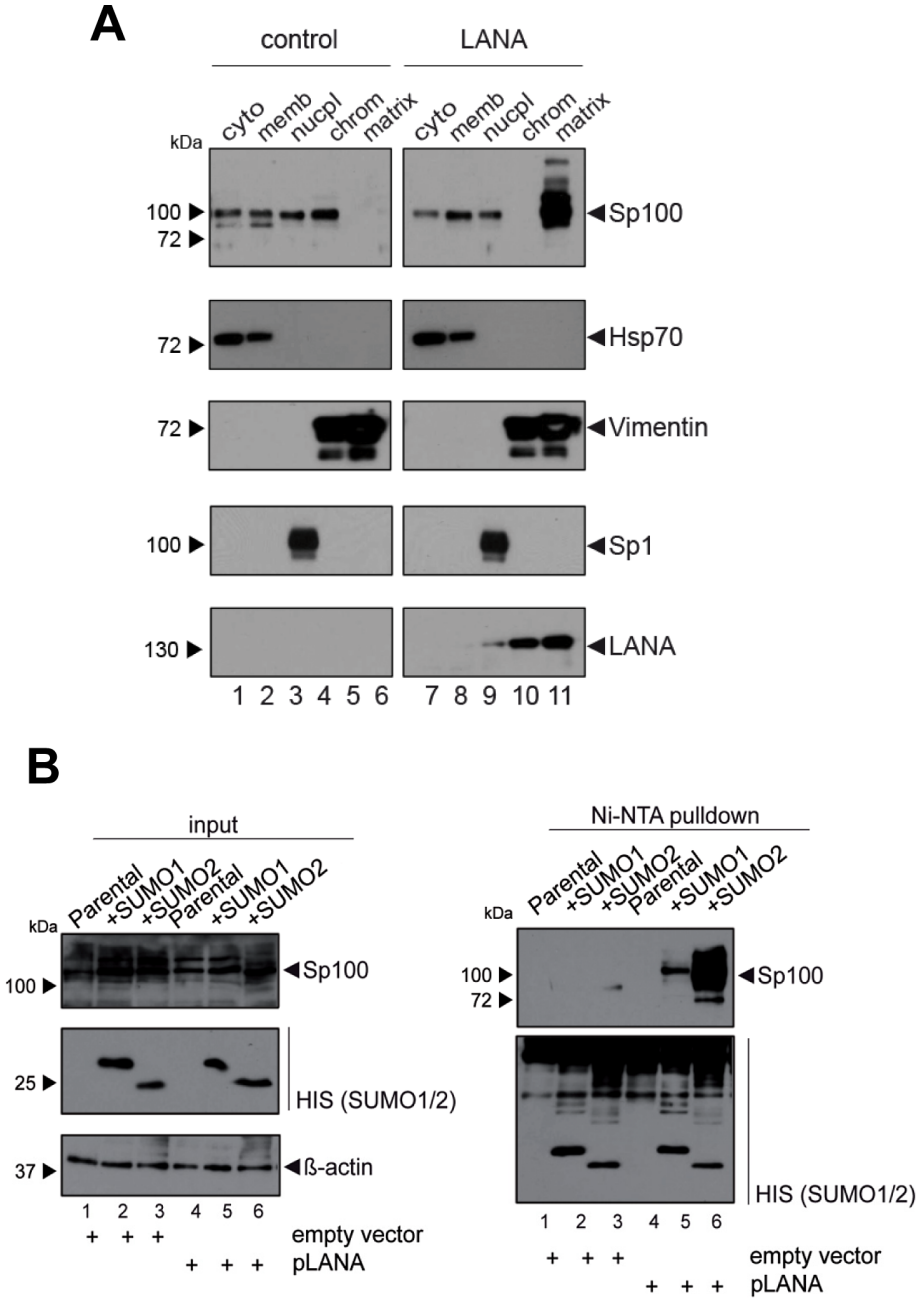
LANA induces relocalization and SUMOylation of Sp100. (**A**) EA.hy cells were transduced with an YFP labeled retroviral LANA expression construct (right panel) or a control retrovirus (left panel). YFP positive cells were enriched by FACS to reduce background of non-transduced cells. After subcellular fractionation, the localization of Sp100 in LANA was analyzed by western blotting. Successful fractionation was confirmed by detection of specific marker proteins (Hsp70, Sp1 and Vimentin). Abbreviations above each lane indicate the following fractions: *cyto*: cytoplasmic; *memb*: membrane-associated; *nucpl*: nucleoplasmic; *chrom*: chromatin-associated; *matrix*: insoluble matrix-associated. (**B**) Stable HIS-tagged SUMO-1 or SUMO-2 expressing and parental HeLa cells were transfected with pcDNA3-LANA or a control vector for 72 hours. Protein lysates were purified via Ni-NTA affinity chromatography and SUMOylated Sp100 or all precipitated SUMOylated proteins were detected by western blot analysis using Sp100 or HIS-specific antibodies (right panel). Input protein levels of Sp100 and HIS-tagged SUMO-1/-2 are shown in the left panel.

Given that fact that Sp100 relocalization requires *de novo* viral gene expression and persists throughout latency, we hypothesized that a constitutively expressed latency factor might orchestrate this phenotype. A particular interesting candidate was LANA, a multifunctional protein which interacts with a number of chromatin factors. Furthermore, analysis of total SLK protein extracts had shown that LANA accumulates precisely when soluble Sp100 levels begin to disappear (Figure 3C). To test whether expression of LANA is sufficient to induce Sp100 relocalization, we generated stably LANA-expressing EA.hy cells by retroviral transduction and analyzed the sub-cellular localization of Sp100 by fractionated western blot analysis (Figure 8A). Indeed, stable expression of LANA cells completely displaced Sp100 from the chromatin associated fraction and relocalized it to the insoluble matrix. As with *de novo* infected EA.hy cells (and in contrast to PEL cells) soluble Sp100 was still present in the nucleoplasmic fraction, supporting the notion that depletion of this fraction may depend on the cellular context or may require extended periods of time.

Since localization of Sp100 to ND10 bodies is modulated by SUMOylation, we hypothesized that LANA exhibits a Sp100-specific SUMOylation-enhancing activity. To directly test this hypothesis, we transfected pcDNA3-LANA or a control vector into HeLa cells that stably express His-tagged SUMO-1 or His-tagged SUMO-2, or the parental cell line (kindly provided by R. Hay). 48 h after transfection, we performed a Ni-NTA pull down to precipitate all proteins that are covalently bound to His-SUMO. SUMOylated Sp100 was subsequently detected by WB analysis with an Sp100-specific antibody (Figure 8B). Indeed, expression of LANA was sufficient to dramatically increase the amount of SUMO-1 and SUMO-2-modified Sp100. Hence, we suspect that massive Sp100 SUMOylation induced by LANA is the major mechanism that drives relocalization of soluble Sp100 during a *de novo* KSHV infection. We furthermore suspect that the matrix-associated entity which receives the relocalized protein may be ND10 bodies themselves, a hypothesis which would be in accord with the observed increase in total number and volume of Sp100 foci at 72 h post infection (Figure 6B), a time point when total Sp100 protein levels are similar to those observed in mock infected cells (Figure 3C). However, at present it remains equally possible that Sp100 may be relocalized to another insoluble matrix fraction.

### Depletion of Sp100, PML or Daxx does not interfere with latency establishment

**Figure 9:**
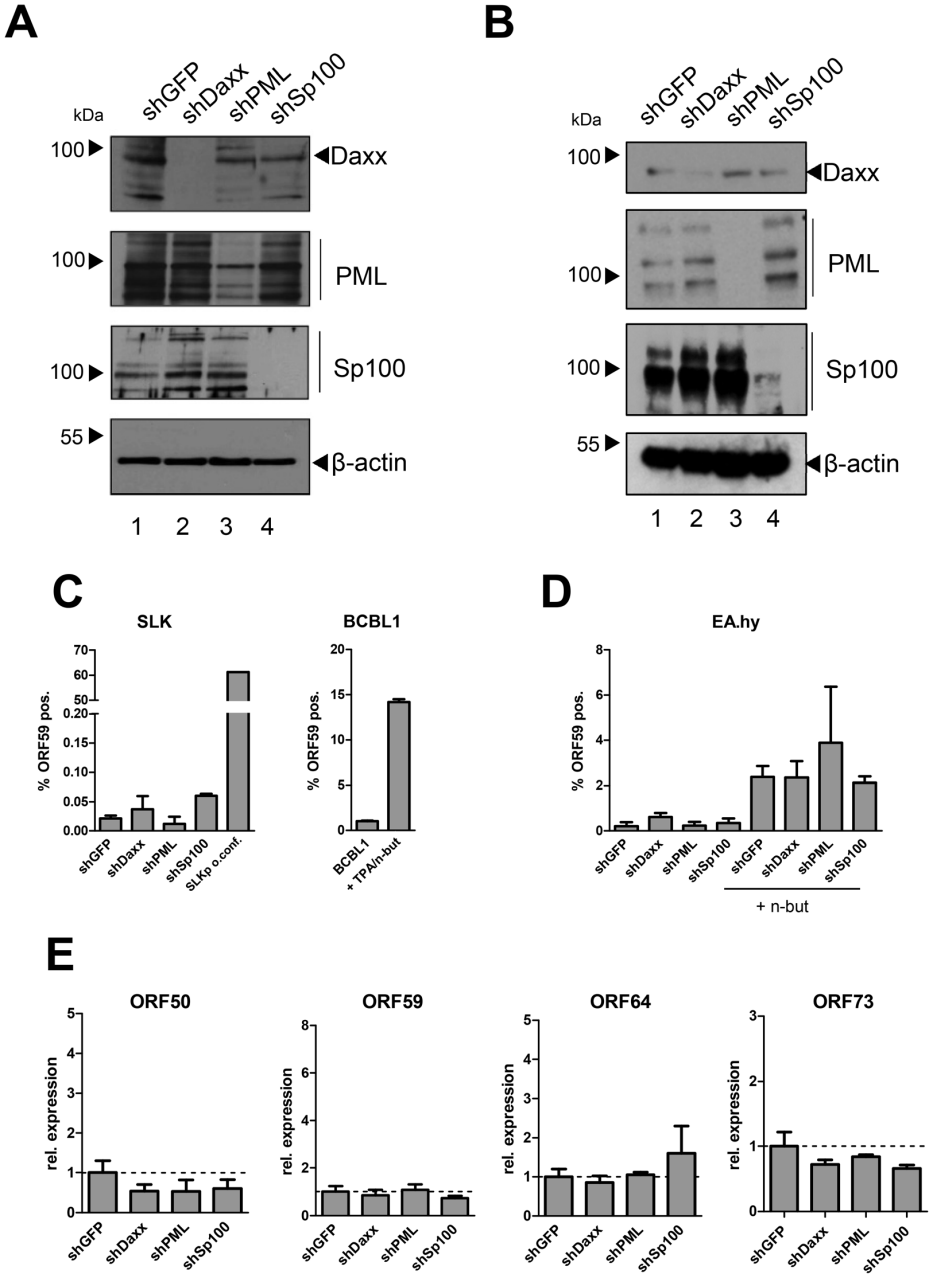
Depletion of ND10 components does not interfere with latency establishment in SLK or EA.hy cells. Cell lines depleted for Sp100, Daxx, PML or GFP (control) were generated by transduction with lentiviruses expressing specific shRNAs. After antibiotic selection, stable SLK (A) and EA.hy (B) cultures were analyzed by western blotting to confirm successful knock down. (**C**): FACS analysis to establish the frequency of ORF59 positive cells in SLK knockdown cultures. The right panel shows a positive staining control employing TPA (20 nM) and sodium butyrate (0.3 mM) induced) BCBL1 cultures 48 h after treatment. Columns 1 to 4 of the left panel show SLK knockdown cultures at 36 h of infection with KSHV. As an additional positive control for ORF59 staining, the rightmost column of the panel shows lytically reactivated cells from long-term infected and overconfluently grown SLKp cultures (see text for details). (**D**): FACS analysis of stably shRNA-expressing EA.hy cultures analysed at 48 hours post infection with KSHV. The rightmost columns show cells which were treated with sodium butyrate (2.5 mM) immediately after infection. Mock infected cells were used to correct for background staining levels in all experiments. Error bars represent SEM of at least two and up to four biological replicates. (**E**) Transcript levels of ORF50, ORF59, ORF64 and ORF73 in EA.hy cells at 48 hours post infection (see Table S1 for RT-qPCR primers). Expression was calculated by normalization to GAPDH and is shown relative to shGFP controls (set to 1). Error bars represent SEM of at least three data sets.

The fact that the major latent nuclear antigen LANA, a protein which has been reported to suppress lytic replication of KSHV [99], [100], induces massive relocalization of soluble Sp100 during a *de novo* infection suggests that this mechanism serves to promote latency. We hypothesized that either the presence of soluble Sp100 may negatively influence latency establishment and is thus actively sequestered by LANA to abrogate such effects (loss of function scenario), or that the SUMOylated and relocalized Sp100 fraction may actively support latency establishment (gain of function scenario). If the latter model were true, then abrogation of Sp100 expression should interfere with latency establishment and result in increased levels of lytic replication upon a *de novo* infection. To investigate this possibility, we stably depleted Sp100 from SLK and EA.hy cultures by using shRNA encoding lentiviruses [101]–[104]. Additionally, we generated cell lines stably depleted for the two ND10 core components PML and Daxx, as well as control cell lines expressing a shRNA directed against GFP. After puromycin treatment to select for shRNA expressing cells, we confirmed depletion of the targeted proteins by WB analysis. As shown in Figure 9A, Daxx and Sp100 were undetectable in SLK cells expressing the respective shRNA. PML levels were substantially reduced, although residual PML protein was still present. In EA.hy cells, levels of PML and Sp100 were most efficiently reduced, whereas a weak band for Daxx was still detectable by western blotting. Immunofluorescence analysis, however, confirmed that the number of Daxx-positive nuclear bodies was nevertheless substantially reduced in shDaxx-transduced EA.hy cells (Figure S6).

To evaluate functionality of the individual knock downs, we infected the SLK cell lines with human Adenovirus (hAdV). In accord with previous findings [104]–[106], depletion of Daxx or Sp100 resulted in an increase of early and late viral gene expression, DNA synthesis and virus yield, whereas PML depletion affected viral gene expression and DNA replication but was nearly dispensable for virus progeny production (Figure S7A, -B and -C, respectively). As for SLK cells, knockdown of Sp100 in EA.hy cells did likewise increase the yield of adenovirus particles (Figure S7D).

After having confirmed functionality of the knock downs, we infected the individual cell lines with KSHV for 36 h. LANA staining confirmed that more than 95% of the cells were infected in each of the cultures (exemplarily shown for EA.hy cells in Fig. S8). To determine the amount of cells containing lytically replicating KSHV, we performed intracellular staining and FACS analysis using an antibody against the ORF59 gene product, a marker for lytic gene expression. Detection of ORF59 in BCBL1 cultures lytically induced by treatment with TPA and sodium butyrate confirmed accuracy of the FACS assay (right panel of Fig. 9C). As an internal positive staining control for SLK cells (which do not well respond to TPA or sodium butyrate treatment), we employed long-term cultures of SLKp cells that had been grown for three weeks without sub-culturing, resulting in lytic cycle induction in a substantial fraction of cells (likely due to hypoxic conditions in basal cell layers; Tessmer and Grundhoff, unpublished). As shown in the left panel of Figure 9C, approximately 60% of the SLK cells in our positive control exhibited positive ORF59 staining. In contrast (and in agreement with our previous findings in SLK cells [14]) the frequency of *de novo* infected SLK-shGFP cells exhibiting ORF59 staining was very low (approx. 0.02%). Interestingly, knock down of the individual core components did not substantially facilitate lytic KSHV replication. Although depletion of Sp100 resulted in a slight increase of ORF59-positive cells (∼0.06%), this observation may be explained by the fact that this population was also slightly more prone to apoptosis (data not shown), a pathway which has been reported to induce lytic replication [107]. As shown in the leftmost columns of Fig. 9D, although EA.hy cells generally exhibited a slightly higher frequency of spontaneously reactivated cells, this frequency was not significantly altered in the knockdown cultures. When treated with sodium butyrate, the percentage of ORF59 positive cells was increased to approximately 2% (rightmost columns of Fig. 9D). Although the average frequency in shPML cells was slightly higher, these cultures exhibited some variability between individual experiments and the differences were not statistically significant. To also directly investigate transcript levels of lytic genes, we performed quantitative RT-PCR for ORF50 (early), ORF59 (delayed early) and ORF64 (late), as well as the latent ORF73 encoding LANA (Figure 9E). In accord with our FACS analysis, the transcript levels for these genes were not significantly altered in the knockdown compared to the shGFP control cultures.

**Figure 10:**
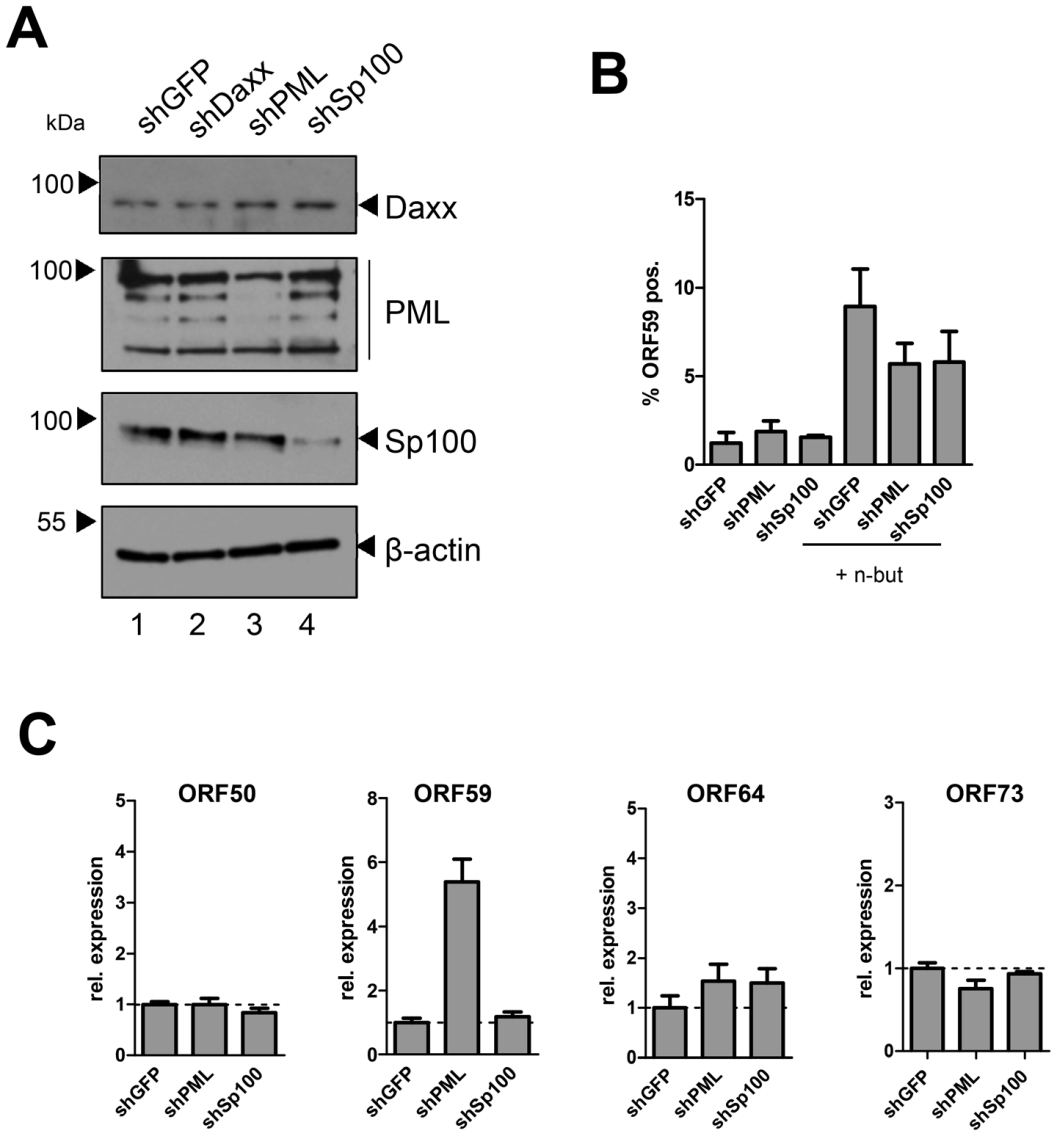
Depletion of PML or Sp100 does not interfere with latency establishment in HUVEC cells. (**A**) Western blot analysis of HUVEC cells transduced with shRNA-expressing lentiviruses directed against PML, Daxx, Sp100 or GFP. shDaxx transduced cells did not show a reduction of Daxx protein levels and were thus not included in further analyses. Although residual levels of PML and Sp100 are still visible in bulk protein extracts, immunofluorescence analysis suggests that a considerable number of cells is devoid of PML or Sp100 positive nuclear bodies (Figure S9). (**B**) FACS analysis of stably shRNA-expressing HUVEC cultures analysed 48 h ater infection with KSHV. The rightmost columns show cells which were treated with sodium butyrate (5 mM) immediately after infection. Mock infected cells were used to correct for background staining levels. Error bars represent SEM of three biological replicates. (**C**) Transcript levels of ORF50, ORF59, ORF64 and ORF73 in EA.hy cells at 48 hours post infection. Expression was calculated by normalization to GAPDH and is shown relative to shGFP controls (set to 1). Error bars represent SEM of at least three data sets.

Lastly, we sought to confirm our results in primary HUVEC cells. Although we were unable to efficiently deplete Daxx, transduction of shRNAs against PML or Sp100 resulted in considerable reduction of protein levels as judged by western blotting (Fig. 10A) or immunofluorescence analysis (Fig. S9). As seen in SLK and EA.hy cells, knockdown of PML or Sp100 did not lead to a significant increase of the frequency of ORF59 positive cells, be it in uninduced or sodium butyrate treated cultures (left- and rightmost columns in Fig. 10B, respectively). Interestingly, we observed a substantial increase in the total levels of ORF59 transcript levels in shPML (but not shSp100) HUVEC cells (Figure 10C). Since the total number of ORF59-positve cells was not increased, we suspect that PML-knockdown, while not interfering with latency establishment, may facilitate ORF59 transcription in the small fraction of cells that spontaneously enter the lytic cycle. Presently, it is unclear why ORF50 and ORF64 levels do not show a similar increase, or why ORF59 transcript levels are not increased in shPML EA.hy cells. Clearly, however, since knockdown of ND10 components did significantly increase the frequency of ORF59-positive cells in SLK, EA.hy or HUVEC cells, our data collectively suggest that neither the presence of intact ND10 bodies, nor that of soluble Sp100, PML or Daxx is a principal requirement for the establishment of KSHV latency and prevention of lytic cycle entry.

### Depletion of Sp100, but not Daxx or PML, accelerates acquisition of repressive chromatin marks on viral episomes

**Figure 11:**
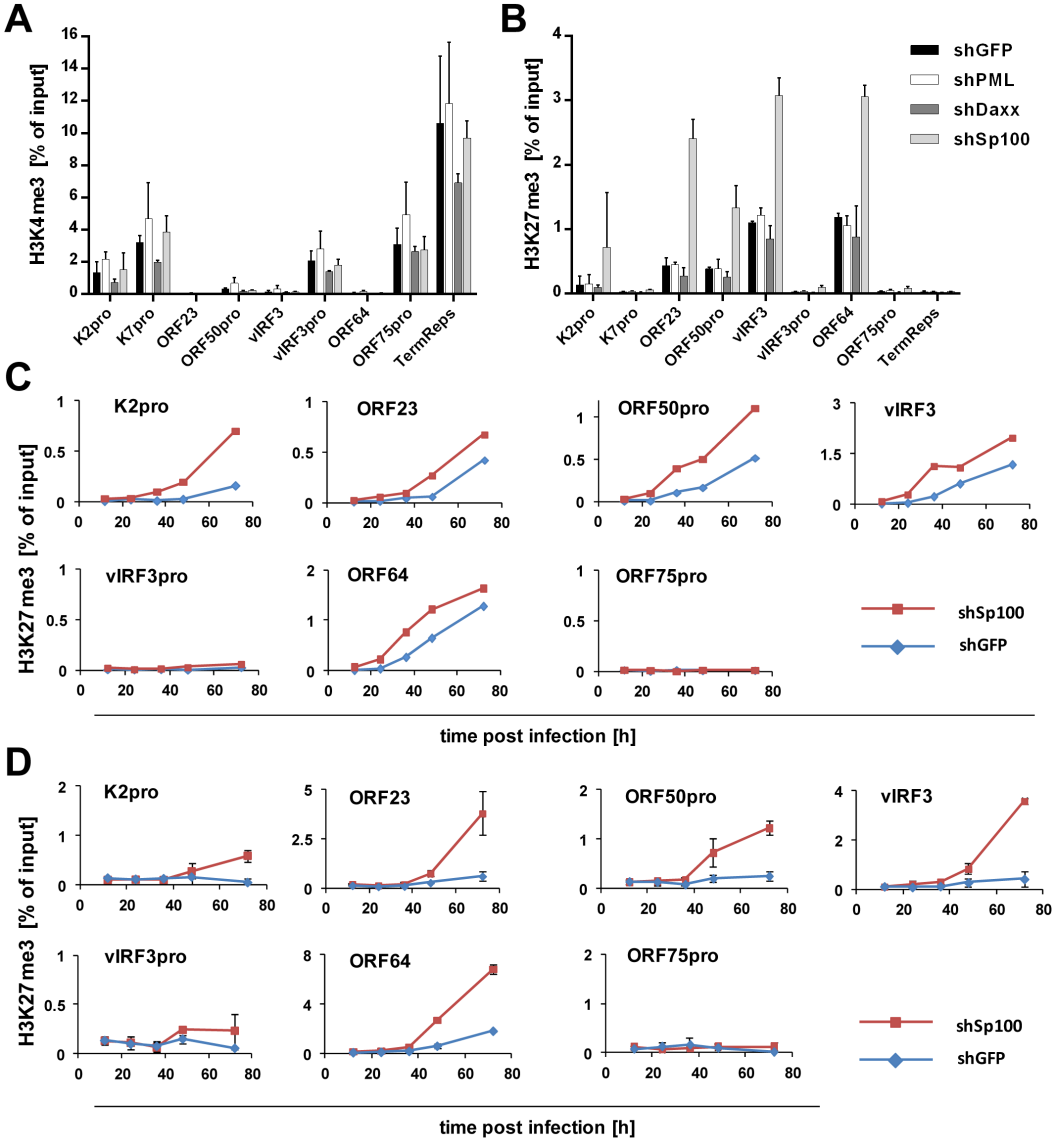
Depletion of Sp100 but not Daxx or PML accelerates acquisition of H3K27me3. (A+B) SLK-shSp100, SLK-shDaxx, SLK-shPML and SLK-shGFP cells were *de novo* infected with KSHV and chromatin was prepared at 36 h post infection. Deposition of activating H3K4me3 (**A**) or repressive H3K27me3 (**B**) marks was evaluated by ChIP-qPCR using specific primers as given in Table S1. (C+D) ChIP-qPCR analysis of selected loci in *de novo* KSHV infected shSp100 (red squares) or shGFP (control; blue diamonds) SLK (**C**) or EA.hy (**D**) cells was performed to analyze deposition of H3K27me3 marks at the indicated time points.

Having ruled out a positive role for relocalized Sp100, we next considered the possibility that the soluble Sp100 fraction may represent a negative factor during latency establishment. Given that Sp100 has been shown to be capable of inducing epigenetic alterations on viral promoters [90], we hypothesized that KSHV actively displaces soluble Sp100 from chromatin to influence the formation of eu- or heterochromatin on viral episomes during latency establishment. Unfortunately, LANA relocalized Sp100 protein with such efficiency that we were unable to increase levels of soluble Sp100 protein by overexpression from an ectopic construct (data not shown). However, since our time course experiments had suggested that the acquisition of heterochromatic H3K27me3 marks coincides with the disappearance of soluble Sp100 protein (Figures 1 and 3), we expected that shRNA depletion of Sp100 may circumvent the need for Sp100 relocalization that occurs only after accumulation of *de novo* expressed LANA protein. If so, then acquisition of heterochromatic marks should be facilitated in *de novo* infected shSp100 knock down cells. To test this hypothesis, we infected the stable SLK-shSp100, SLK-shPML and SLK-shDaxx knock down cell lines and the control cell line (SLK-shGFP) with KSHV for 36 h and subsequently performed ChIP using antibodies against activating (H3K4me3) and inactivating (H3K27me3) histone modifications. We then measured the enrichment of the respective modification by qPCR at a number of selected loci that predominantly carry activating or repressive marks in latently infected SLK cells. As shown in Figure 11A and -B, the overall pattern for either modification was not altered in SLK-shGFP when compared to those seen in our initial ChIP analysis at early time points of infection (Figures 1 and S1). Depletion of PML or Daxx resulted in only a slight (but nevertheless consistent) increase or decrease, respectively, of H3K4me3 levels compared to the control, whereas H3K27me3 levels remained unaffected. In contrast, depletion of Sp100 dramatically increased H3K27me3 levels, but did not alter levels of H3K4me3 at loci which do not carry this repressive mark. The fact that overall patterns of activating and repressive marks remained unaltered suggested that Sp100 depletion results in an enhanced rate of H3K27me3 acquisition, but not a qualitative change of the underlying recruitment process. To further confirm these findings, we verified the increased H3K27me3 in shSp100 cells by ChIP-qPCR in a time-course experiment. As shown in Figure 11C, acquisition of repressive chromatin marks was indeed highly accelerated in Sp100-depleted cells. To exclude cell type specific effects, we additionally investigated H3K27me3 acquisition in Sp100-depleted EA.hy cells. Interestingly, while overall the patterns of H3K27me3 in shGFP-EA.hy control cultures were very similar to those observed in shGFP-SLK cells, the repressive mark evolved more slowly in the EA.hy cells (compare blue graphs in Figure 11C and -D). This observation may be explained by the fact that Sp100 is displaced with delayed dynamics in KSHV infected EA.hy cells when compared to SLK cultures (Figures 3B and 4B, respectively). In agreement with this hypothesis, and in perfect accord with the observation made in SLK cells, depletion of Sp100 dramatically accelerated acquisition rate, but not overall patterns of H3K27me3 (Figures 11D and S10) in shSp100-EA.hy cultures.

## Discussion

ND10 bodies have been linked to the intrinsic immunity which counteracts productive replication of different viruses [49]–[51], [56], [57], [108]. Conversely, these viruses have evolved mechanisms to counteract repressive capabilities of individual ND10 components [108]. Since ND10 components exhibit chromatin modulatory functions [109] and can cause epigenetic alterations on viral promoters [90], it has been speculated that ND10 components may also be able to impose a repressive chromatin state that could potentially represent a necessary - or even sufficient - event during herpesvirus latency establishment. In such a scenario, ND10 bodies would serve as interferon-induced host restriction factors that are universally recruited to incoming viral DNA genomes, e.g. via recognition of unmethylated DNA. Normally, ND10 recruitment would serve to globally silence and, ultimately, eradicate the virus. However, herpesviruses may have subverted this mechanism by allowing transient silencing while preventing elimination of viral episomes. *Bona fide* latency may then ensue if the repressive state becomes self-sustaining, a condition which is met by a number of repressive chromatin marks, including the H3K27me3 modification that is a hallmark of KSHV latency [19]–[22].

We here report the first systematic study of the role of ND10 bodies as well as individual ND10 components on the establishment of KSHV latency. Although we uncover an unexpected role for soluble fractions of Sp100 (discussed further below), our data collectively suggest that silencing activities of intact ND10 bodies in fact only play a minor, if any, supportive role during the establishment of primary KSHV latency. In accord with previous studies reporting cytokine responses of primary cells to latent KSHV infection [reviewed in 110], [111], we find that *de novo* infection of SLK cells results in a marked (but brief) induction of cytokines and subsequent transcriptional upregulation of Sp100, PML and other interferon response genes. However, immunofluorescence analysis strongly suggests that KSHV episomes do not reside near ND10s at the time when repressive H3K27me3 marks are first acquired, and also do not communicate with these bodies during later phases of viral latency. This conclusion is based on the fact that foci of the episome-binding LANA-protein do not co-localize with PML or Sp100 speckles during the first 24 to 72 hours of a *de novo* infection. LANA foci have been previously shown to accurately mark the location of episomes in latently infected cells [10], [94], and we have confirmed perfect co-localization between LANA and viral episomes in SLK cells at 72 h post infection (Figure S2). Given that the focal accumulation of LANA is induced by high-affinity binding to the terminal repeat elements of viral episomes and represents a pre-requisite for latent replication of KSHV genomes [11]–[13], it is very likely that the LANA foci observed at 24 or 48 h post infection do likewise contain viral DNA, even though we have not explicitly investigated these time points by IF/FISH analysis. It may be argued, however, that especially at earlier time points of infection a sub-fraction of viral episomes may not be complexed with LANA and could potentially interact with ND10s. Of course, this could be especially true during the first few hours of an infection, before *de novo* expressed LANA protein has accumulated to sufficient amounts. In fact, given ND10s role as antiviral factors we consider it probable that such early events may indeed occur and could lead to terminal silencing (and subsequent elimination) of a considerable fraction of incoming KSHV episomes. However, for the following reasons we find it unlikely that such episomes could also represent a founder population of KSHV latency: Firstly, we observe acquisition of the repressive H3K27me3 mark between 24 and 72 h post infection, at a time when LANA is well expressed and localizes in episome-containing speckles that are distinct from ND10s. Since the histone modification patterns that evolve during this time are precisely those which are observed in long term latently infected cells, there is every reason to believe that it is this population of episomes which goes on to establish latency. More importantly, however, knockdown of the ND10 components PML, Daxx or Sp100 did not interfere with latency establishment. We did not observe any increase in the percentage of KSHV-infected cells undergoing lytic replication, or any delay in the acquisition of repressive H3K27me3 marks by KSHV episomes. We point out that these experiments were carried out at a high multiplicity of infection (MOI), leading to the establishment of latency in more than 90–95% of cells. In systems such as HSV or CMV, interference with productive infection is often only observed when low MOIs are used, arguing that high levels of viral genomes can readily outcompete suppressive functions of ND10s. By the same token, if ND10-mediated repression mechanisms were indeed a pre-requisite for KSHV latency, at the high MOI levels used here we would expect that even a modest knockdown of one of the ND10 core components would allow at least some viral genomes to escape ND10s and proceed to enter the lytic cycle. As this is not the case, we consider it very unlikely that ND10s serve as nucleators of KSHV latency.

Instead of intact ND10 bodies, our study has uncovered an unexpected role of soluble Sp100 during latency establishment (strictly speaking, this fraction should be referred to as ‘solubilizable’ Sp100 since it involves nucleoplasmic as well as low-salt extractable chromatin-bound protein; for simplicity, however, we have referred to it as the soluble fraction throughout this manuscript). We consistently observed that this fraction was efficiently relocalized to the insoluble matrix between 24 and 72 hours post infection, i.e. precisely the time period when repressive H3K27me3 marks accumulate on viral episomes. This phenotype is likely mediated by *de novo* expressed LANA protein, given that ectopic expression of LANA alone is sufficient to induce relocalization and massive SUMOylation of Sp100. Consistent with this hypothesis, we observe that UV-irradation of virion preparations prevents the disappearance of Sp100 from soluble fraction, and that accumulation of LANA protein in de novo infected SLK cells is concomitant with the disappearance of soluble Sp100. Interestingly, LANA has been demonstrated to exhibit SUMOylation-enhancing activity towards histone proteins [112], and was very recently reported to contain a SUMO-interacting motif (SIM) that is specific for SUMO-2 [113]. The latter study further demonstrated that LANA itself is SUMO-2-modified, a phenotype which is dependent on the presence of its SIM domain. Strikingly, a recombinant virus with a mutant LANA SIM was unable to establish latency and exhibited severely reduced lytic gene silencing [113]. Given that LANA-induced SUMOylation of Sp100 also shows a marked preference for SUMO-2, we suspect that the LANA SIM is also required for Sp100 modification, and that furthermore inability to induce Sp100 relocalization may have contributed to the latency deficiency observed in the SIM mutant virus investigated by Cai and colleagues [113]. Our results extend the SUMO targetome of LANA by Sp100, and it appears likely that further targets may exist. Interestingly, the master lytic switch protein Rta has been recently shown to exhibit SUMO-targeting ubiquitin ligase (STUbL) activity, resulting in ubiqitinylation and subsequent degradation of SUMOylated proteins bound to the Rta SIM domain [114]. This may hint towards the existence of novel protein regulatory networks in which LANA may create a set of SUMOylated proteins to support latency, which in turn can be readily targeted for degradation by Rta once the lytic cycle has been induced. Whether or not such a mechanism indeed exists is currently under investigation.

Since SUMOylation has been shown to facilitate protein recruitment to ND10s we suspect that massive accumulation of Sp100 in the insoluble matrix is indicative of its relocalization to these bodies. This is consistent with the observation that Sp100 foci are increased in number and volume at 72 h post infection, at a time point when the (likely interferon induced) mild increase of total Sp100 protein levels has abated. We cannot fully exclude the possibility, though, that the protein may instead be diffusely redirected to some other part of the matrix, in a manner that precludes its detection by immunofluorescence analysis. However, considering the extent of relocalization especially in PEL cells we find this scenario rather unlikely. In accord with previous studies [98], [115], we find that also a sizeable fraction of LANA protein is associated with the insoluble matrix. Since this fraction has previously been found to associate with viral episomes it is likely to be spatially distinct from relocalized Sp100 protein. This notion is also supported by the fact that we did not observe any significant co-precipitation of LANA and Sp100 (data not shown), indicating that any putative direct interaction is likely to be transient and restricted to the SUMOylation process.

**Figure 12:**
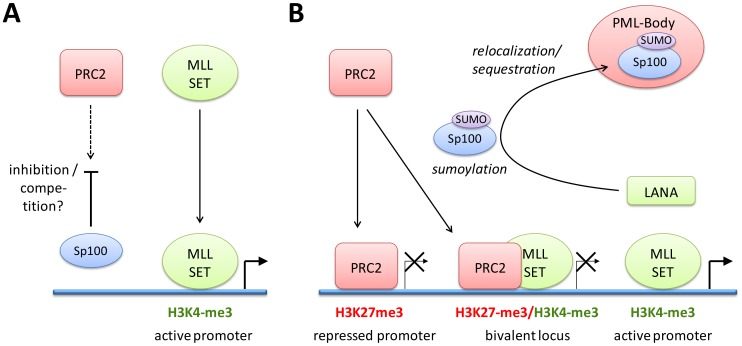
Model of events during early KSHV latency establishment. (**A**) Immediately after infection, MLL/SET containing complexes are recruited to viral episomes by as of yet unknown cellular and/or viral factors, resulting in the establishment of the early activation marks and transcription of a subset of KSHV genes that includes ORF73/LANA. Recruitment or activity of PRC2 to KSHV episomes is inhibited by the presence of soluble Sp100. (**B**) After accumulation of *de novo* expressed LANA protein, LANA induces SUMOylation and relocalization of Sp100 into insoluble fractions which may be ND10s, but could also represent another matrix-associated fraction. Depletion of soluble nucleoplasmic or chromatin-associated Sp100 allows widespread recruitment of PRC2 complexes via molecular mechanisms that remain to be determined. PRC2 recruitment establishes H3K27me3 patterns, allowing repression of lytic genes after extinction of activation marks (e.g., on the K2 promoter) or establishment of bivalent chromatin (e.g. at the ORF50/Rta promoter). The major latency promoter upstream of ORF73/LANA is protected from H3K27me3 acquisition and remains active throughout latency.

Our infection studies of Sp100 knock down cells strongly argue for the hypothesis that soluble Sp100 hinders latency establishment rather than suggesting a latency promoting activity of the relocalized protein. Based on the observations made in this study, we propose a model as depicted in Figure 12: After nuclear delivery of viral episomes, the DNA becomes rapidly occupied by histones, but remains accessible to the binding of factors that facilitate the site-specific acquisition of H3K4me3 marks by recruitment of MLL/SET protein complexes. Such factors are likely to include host transcription or other chromatin-modifying factors such as CTCF, but may also include virally encoded factors - the finding that transfected KSHV bacmids ultimately carry the same H3K4me3 profile as in infected cells does not preclude the possibility that their acquisition may be accelerated by virion-delivered proteins. The acquisition of these early activation marks then allows a ‘relaxed’ transcription program that includes LANA, but also a limited set of other genes that are usually not expressed during latency [116]. We propose that during this immediate phase, i.e. before accumulation of LANA protein, deposition of repressive H3K27me3 marks is hindered by soluble Sp100 protein. The mechanisms by which Sp100 may prevent this process remain unknown, but could potentially include negative regulation of the catalytic activity of PRC2 complexes or interference with PRC2 recruitment. We find the latter possibility particularly attractive since LANA preferentially depletes the chromatin bound Sp100 fraction, and since furthermore the SAND domain of the SP100B splice variant has been shown to exhibit broad binding activity towards unmethylated DNA [117]. Given that CpG methylation is completely absent from *de novo* infecting episomes [14], it seems possible that non-ND10 resident Sp100 may be rapidly attracted to KSHV episomes and may either competitively block PRC2 recruitment, or sequester viral genomes in subnuclear regions that are not readily accessible to PRC2. LANA depletion of chromatin-bound Sp100 then serves to alleviate this effect, allowing PRC2 recruitment and establishment of ‘mature’ latent chromatin characterized by the distinct patterns of predominantly H3K27me3-, H3K4me3-, or bivalently marked regions as shown in Figure 1.

Collectively, our data suggest that KSHV does not depend upon ND10-mediated silencing to establish latency, but rather actively escapes such mechanisms in order to promote a chromatin state that is more amenable to latent replication and reactivation of the lytic cycle. Notably, this model does not exclude the possibility that innate immune responses do actively contribute to latency establishment. It is entirely possible that PRC recruitment reflects an alternate host defense silencing mechanism that come into effect once first line ND10 defenses have failed to efficiently repress viral DNA. By removing chromatin-associated Sp100 fractions, KSHV may skew host defenses towards the preferred formation of facultative heterochromatin by polycomb repressive complexes

Whether this strategy is uniquely employed by KSHV or shared by other herpesviruses remains to be established. Clearly, the data shown in Figure 6B demonstrate that EBV does not relocalize soluble Sp100 to the matrix in latently infected BL-cell lines. This finding is in accord with a previous study which reported that the latent EBNA-LP protein displaces Sp100 from ND10s and relocalizes it to the soluble fraction [118]. Furthermore, a recent study reported that EBV latent membrane protein 1 (LMP-1) can induce expression of the H3K27me3 specific demethylase KDM6B [119], indicating that EBV may have evolved a fundamentally different approach to establish latency. In line with this notion, we have observed that EBV episomes carry fundamentally lower levels of H3K27me3 compared to KSHV (Guenther et al., unpublished observations). A question that emerges from these observations is whether EBV and KSHV may mutually influence each other on the chromatin level. This is a particular interesting scenario since the great majority of PELs carry both viruses, and since EBV shows a non-typical latency expression pattern in dually infected PEL-derived cell lines as well as primary PEL tissues [120], [121]. We have observed that dually infected cells do not differ from KSHV-only infected PEL in their exclusive matrix-association of Sp100 protein (Figure 6B). Whether this is simply due to the fact that dually infected PEL cells do not express EBNA-LP (and only low levels of LMP-1) [120], [121], or whether LANA may be able to reverse EBNA-LP functions remains to be established. We are currently investigating whether such a mutual influence indeed exists.

The temporally ordered acquisition of activating H3K4me3 and H3K27me3 marks as observed here suggests that KSHV transits from an open chromatin state to one that becomes progressively repressed. Unfortunately, our RNAseq experiments, although carried out at a standard depth of approximately 20 million mapped paired-end reads per sample, were not sensitive enough to allow reliable comparison of global viral transcription patterns to the evolving histone modification landscape. On average, only about 800 reads per sample (i.e. less than 0.005% of all mapped reads) were of viral origin. The only viral genes that produced significant expression values were PAN, K2, and the ORF73/72/71 latency cluster (Figure S11). In accordance with the observation that activation-associated H3K4me3 marks were prominently present in the K2 region at 24 h post infection, but were progressively diminished upon accumulation of H3K27me3 (see leftmost arrow in Figure 1B), transcript levels for K2 peaked between the 16 to 24 h RNA-seq time points and sharply declined thereafter. In contrast, transcripts from the major latency ORF73/72/71 locus remained fairly stable after the 24 h time point. Interestingly, we observed a transient peak of ORF73/72/71 transcription at 8 h post infection. The significance of this observation is currently unknown, however we noted that at least one interferon-inducible cellular factor known to transactivate the ORF73/LANA promoter was sharply upregulated at 4 h post infection (HIF1A; see Figure S11, lower panel). Hence, the immediate response may help to ensure early transcription of ORF73/LANA.

Considering that the viral genome length is about 0.2% of the combined exon length of all expressed host transcripts (∼55.000 kbp), the fact that less than 0.005% of all reads originated from KSHV indicates that the remainder of the viral genome is transcribed at considerably lower levels than host genes, even though the episome is in a mostly open chromatin configuration during the early stages of infection. More detailed RNA-seq experiments will be required to investigate this issue. However, if so, it seems possible that either much of the early viral chromatin is ‘immature’ and does not allow effective transcription even in the absence of repressive marks, or that other factors may contribute to repression at this stage. Interestingly, while this study was being prepared for submission, Toth et al. [18] reported results which confirm the biphasic acquisition of H3K4me3 and H3K27me3 histone marks as shown in Figure 1B. While Toth and colleagues show that knockdown of PRC2 components considerably lowers PRC1 recruitment and enhances viral transcription of a number of lytic genes, some loci were found to attract PRC1 complexes even before H3K27me3 marks had evolved, indicating that direct recruitment of PRC1 may also contribute to transcriptional regulation of early viral chromatin.

An important question that remains to be answered is how the KSHV genome initially attracts polycomb repressive complexes. In insect cells, polycomb repressive complex bind to DNA sequence elements (polycomb response elements; PREs), yet the mechanisms that lead to polycomb recruitment in mammalian are poorly understood. The fact that Sp100 knock down accelerated the acquisition of the H3K27me3 mark without altering its overall patterns suggests that Sp100 does not modulate PRC2 recruitment in a qualitative manner. Hence, our study suggests that LANA-mediated depletion from Sp100 primarily serves to allow attraction of polycomb repressive complexes, presumably by an inherent feature of viral genomes or early viral chromatin. Consistent with the notion that mechanisms other than PREs may mediate polycomb recruitment in mammalian cells, our temporal investigation as shown in Figure 1B clearly indicates that H3K27me3 does not spread from initial nucleation sites, but gradually evolves over the entire episome. Features such as absence of DNA methylation, biased histone variant deposition, production of non-coding RNAs or the presence of stalled RNA Polymerase on immature viral chromatin are all potential factors that could contribute to initial recruitment of polycomb repressive complexes [24]–[31], [122], [123]. To what extent these factors contribute to KSHV latency, and whether or not KSHV may also actively modulate one or more of such mechanisms will be interesting topics for future studies. We expect that the results from such studies will not only fundamentally advance our understanding of latency establishment by KSHV and other herpesviruses, but will also allow valuable insights into as of yet poorly understood mechanisms of cellular chromatin regulation pathways.

## Materials and Methods

### Cell lines and culture conditions

SLK [124], SLKp [91], EA.hy [125], human dermal fibroblasts (HDF), HEK293 [126] and HeLa cells were cultured in DMEM supplemented with 10% fetal calf serum and penicillin-streptomycin (5 µg/ml). Human umbilical vain endothelial cells (HUVEC) (PromoCell C-22010) were cultured in EBM-2 Medium including supplements (LONZA). The KSHV-positive PEL cell line BCBL1 [127], the KSHV/EBV-positive cell lines HBL6 [128] and CRO-AP/2 (AP2) [129], the EBV-positive Jijoye [130] and Raji [131] cell lines and the KSHV/EBV-negative BJAB cell line [132] were cultured in RPMI 1640 medium (Invitrogen) supplemented with 10% fetal calf serum and penicillin/streptomycin at a final concentration of 5 µg/ml. All cells were cultured in a 5% CO2 atmosphere at 37°C.

### Viruses, infection and transduction

*KSHV and infection:* Concentrated supernatants of infectious KSHV virions were harvested from lytically induced BCBL1 cells as described previously [91]. *De novo* infection of SLK, EA.hy, HUVEC and HDF was performed by incubating 2×10^5^ cells at 70% confluency for 2 h with 600 µl virus supernatant at a concentration of approximately 1×10^8^ KSHV genome equivalents per ml (as determined by quantitative PCR) in the presence of 8 µg/ml polybrene in EBM-2 medium (Lonza). Generally, more than 95% of cells were infected, as judged by immunofluorescence analysis for LANA 48 h after infection. For UV crosslinking of KSHV prior to infection virus-containing supernatant was exposed to 1200 µJoules UV light for four times using a UV Stratalinker 1800 (Stratagene). These settings were determined to result in approximately 1% LANA-positive cells after 48 h of infection as determined by IF analysis. *Retroviruses:* LANA coding sequence was cloned into the retroviral pMYs-iV vector [133]. Pseudotyped infectious retroviral particles were produced by cotransfecting retro-LANA, VSVG and gag/pol-containing vectors into HEK293 cells. Supernatants were harvested 48 and 72 h post transfection. *Lentiviruses:* Lentiviral constructs encoding shRNAs directed against Sp100, Daxx, PML and GFP have been described before [101]–[104]. Production of infectious lentiviral particles was achieved similar to retroviruses, by co-transfection of lentiviral constructs with VSVG, gag/pol and rev expressing vectors into HEK293 cells. *Transduction:* SLK and EA.hy cells were transduced with recombinant retro-/lentiviruses by spin inoculation at 300× g for 1 h, using undiluted supernatants in the presence of 8 µg/ml polybrene. After inoculation, lentivirus treated cultures were maintained in medium containing 0.2 µg/ml puromycin to select for transduced cells whereas retrovirus treated LANA expressing and control cells were enriched according to YFP expression by FACS. HUVEC cells were transduced for 2 hrs similar to SLK and EA.hy cells, but without spin inoculation. After 48 hrs, transduced cells were selected for an additional 48 to 72 hrs using 0.5 µg/ml puromycin, followed by infection with KSHV for downstream analyses.

### RIPA-extracts, subcellular fractionation and western blot analysis

All buffers described in this section used for protein extraction contained 1% (v/v) PMSF, 0.1% (v/v) aprotinin, 1 µg/ml leupeptin, 1 µg/ml pepstatin) to inhibit proteases. For protein analysis cells were resuspended in full RIPA lysis buffer (50 mM Tris-HCl/pH 8.0, 150 mM NaCl, 5 mM EDTA, 1 mM DTT, 0.1% sodium dodecyl sulfate, 1% Nonidet P-40, 0.1% Triton X-100, 0.5% sodium deoxycholate) as described previously [105]. After 1 h on ice, the lysates were sonicated for 30 sec (total cell lysate); alternatively we pelleted and discarded the insoluble cellular debris (14000 rpm/4°C; soluble lysate). Alternatively, cells were fractionated based on a protocol described by Leppard et al. [134] with some modifications: Cells were resuspended in buffer IB (10 mM Tris-HCl/pH 7.5, 150 mM NaCl) with 10% Nonidet P-40 on ice and pelleted at 1000 rpm/4°C (supernatant contains cytoplasmic fraction). Nuclei were washed with IB and resuspended in IP (10% Nonidet P-40, 10% sodium desoxycholat (SDC)) and pelleted at 1000 rpm/4°C (supernatant contains nuclear membrane fraction). Pellets were resuspended with RSB buffer (10 mM Tris-HCl/pH 7.5, 10 mM NaCl, 3 mM MgCl_2_, 1% (v/v) PMSF, 0.1% (v/v) aprotinin, 1 µg/ml leupeptin, 1 µg/ml pepstatin) containing DNaseI and incubated for 30 min, pelleted at 1200 rpm/4°C (supernatant contains soluble nuclear fraction). Chromatin was dissolved by adding RSB buffer and 5 M NaCl and centrifugation at 2000 rpm/4°C (supernatant contains chromatin fraction). Sediment was resuspended in F5B (10 mM EDTA, 0.2% sodium dodecyl sulfate, 10 mM Tris-HCl/pH 7.5, 150 mM NaCl, 1% (v/v) PMSF, 0.1% (v/v) aprotinin, 1 µg/ml leupeptin, 1 µg/ml pepstatin) to recover the insoluble nuclear and cytoskeletal matrix fraction.

For western blot analysis of steady state protein levels, solutions containing equal amounts of total protein were mixed with an appropriate volume of SDS sample buffer, boiled for 3 min at 95°C, separated by standard SDS-PAGE, transferred to nitrocellulose membranes (Schleicher & Schüll/Whatman) and incubated as described previously [135]. Proteins were visualized by enhanced chemiluminescence (ECL) as recommended by the manufacturer (Pierce) on X-ray films (CEA RP, medical X-ray film). Autoradiograms were scanned and cropped using Adobe Photoshop CS6 figures were prepared using Adobe Illustrator CS6 software and Microsoft Office Power Point 2008/2010. Primary antibodies specific for cellular proteins included anti-Sp100 rabbit polyclonal GH3 [136], anti-Daxx rabbit polyclonal antibody (Upstate/Millipore: #07-471), anti-PML rabbit polyclonal Ab (Novus Biologicals: H-238), anti-Vimentin V9 mouse monoclonal Ab (Sigma-Aldrich, Inc), anti-HDAC2 3F3 mouse monoclonal Ab (Upstate/Millipore), anti-Sp1 (PEP2) rabbit polyclonal (Santa Cruz), anti-Hsp70 mouse monoclonal (Stressgen), anti-β-actin mouse mab AC-15 (Sigma-Aldrich, Inc.) and polyclonal rabbit anti-LANA. Secondary Ab conjugated to horseradish peroxidase (HRP) to detect proteins by immunoblotting were anti-rabbit IgG, anti-rat IgG and anti-mouse IgG (Jackson/Dianova).

### Denaturing purification and analysis of SUMO conjugates

HeLa cells were transfected with either pcDNA3/73 [137] (pLANA) or pcDNA3 (empty vector). Cells were lysed 48 h post transfection in 5 ml 6 M Guanidinium-HCl, 0.1 M Na_2_HPO_4_, 0.1 M NaH_2_PO_4_, 10 mM Tris-HCl pH 8.0, 20 mM Imidazole and 5 mM β-Mercaptoethanol [138]. 500 µl lysate was precipitated with 5× SDS buffer for total protein analysis. Lysates were incubated 12 h at 4°C with 25 µl Ni-NTA agarose (QIAGEN) prewashed with lysis buffer. The slurry was washed with lysis buffer; then with Buffer A (8 M urea, 0.1 M Na2HPO4, 0.1 M NaH2PO4, 10 mM Tris-HCl pH 8.0, 20 mM Imidazole and 5 mM β-Mercaptoethanol) and with buffer B (8 M urea, 0.1 M Na2HPO4, 0.1 M NaH2PO4, 10 mM Tris-HCl pH 6.3, 20 mM Imidazole and 5 mM β-Mercaptoethanol). After denaturation, proteins were separated by SDS-PAGE, transferred to PVDF and visualized by immunoblotting.

### Chromatin immunoprecipitation (ChIP) and qPCR

ChIP analysis was performed as described in Günther and Grundhoff (2010) [14]. Briefly, chromatin from approximately 1×10^6^ cells was cross-linked using 1% formaldehyde. The reaction was quenched by addition of glycine and nuclei were isolated to reduce background. Chromatin was extracted and fragmented by sonication (Bioruptor, Diagenode) to an average length of 100–500 bp. 1/3^rd^ of the prepared chromatin was subjected to each immunoprecipitation using 4 µg of antibodies specific for the histone modifications H3K4me3 (Millipore: #04-745), H3K27me3 (Millipore: #07-449) or normal rabbit IgG (Millipore: #12-370). Chromatin-immunocomplexes were precipitated after 16 h at 4°C using protein-A agarose beads, Bead bound complexes were washed, eluted and de-crosslinked overnight at 65°C. DNA was subsequently purified by phenol-chloroform extraction and ethanol precipitation. Input controls (1/4^th^ of the amount of chromatin used in each immunoprecipitation reaction) were treated in an identical manner as the immunoprecipitated samples, starting with overnight de-crosslinking. Samples and inputs were analyzed either by ChIP on microarray analysis as described in Günther and Grundhoff (2010) [14], or by qPCR using SensiMix SYBR kit (Bioline) and Rotor-Gene Q (QIAGEN) with the primers given in Table S1.

### Immunofluorescence (IF) and flow cytometry

*IF:* Adherent cells were fixated on cover slips using ice-cold methanol for 10 min at RT. Samples were dried, rehydrated in PBS, blocked with 3% BSA/PBS and incubated with primary antibodies rabbit anti-LANA, rat anti-Sp100 [53], rabbit anti-Sp100 (GH3) [136] and mouse anti-PML (Santa Cruz) in blocking solution for 2 h. Cells were washed five times with PBS and incubated with secondary antibodies (Alexa Fluor-555 goat anti-mouse and Alexa Fluor-488 goat anti-rabbit) for another 2 h. After five final washes in PBS, samples were embedded in VECTASHIELD mounting medium with DAPI (Vector Labs) and analyzed by conventional or confocal fluorescence microscopy. *Intracellular IF staining and flow cytometry:* To measure the percentage of lytically reactivated cells within a KSHV infected population, cells were detached from the culture dish using trypsin (PAA) and fixated with 4% paraformaldehyde (PFA) in PBS for 20 min at RT. Fixated cells were washed one time in permeabilization buffer (2% FCS, 0.5% saponine and 0.2% sodium azide in PBS) and two times with superpermbuffer (permeabilization buffer+1 part FCS). Staining was performed using primary antibody against ORF59 (Advanced Biotechnologies: #13-211-100) in superpermbuffer for 30 min at RT. Cells were washed twice with permeabilization buffer and incubated with secondary antibody (Alexa-555 or Alexa-488 anti-mouse) in superpermbuffer for 30 min at RT. After two additional wash steps in permeabilization buffer, cells were resuspended in staining buffer (permeabilization buffer without saponine) and analyzed by standard flow cytometry. Due to higher autofluorescence found in EA.hy and HUVEC cells, these cultures were fixated in 1% formaldehyde and permeabilization was achieved using Triton-X100 prior to staining with buffers omitting saponine.

### Image processing and dot measurement

Confocal microscopic images of IF analyses were generated using an Eclipse T*i* microscope (Nikon). Images are presented as maximum intensity projections (MIP) of z-stacks throughout the nucleus (z step-size: 250 nm). MIPs were created using Imaris (Bitplane). DAPI and PC images represent one confocal slice. For dot measurement, nuceli of >60 cells were imaged using identical microscope settings. Sp100-containing dots were identified and measured per nucleus after 3D reconstruction using Volocity 6.3 (Perkin Elmer). Detection thresholds were determined automatically by standard deviation settings in Volocity. The total volume of dot-associated Sp100 was calculated as the sum of volume of all dots within each individual nucleus that met the threshold criteria. Bars in Sp100 related dot or volume graphs represent mean and SEM. *P*-values were generated by unpaired t-test using Prism 6 (GraphPad).

### RNA isolation and RT-qPCR

RNA was isolated using the RNA-Bee (Tel-Test, Inc.) reagent. Contaminating DNA was removed by incubation with amplification grade DNase I (Invitrogen) and cDNA was prepared from random-primed RNA using Superscript III (Invitrogen) as per the manufacturer's instructions. Real-time quantitative PCR (qPCR) of cDNA or genomic DNA samples was performed using SensiMix SYBR Kit (Bioline) on a Rotor-Gene Q light cycler (QIAGEN). For quantitation, standard curves were created using dilutions of genomic BCBL1 DNA over a range of at least 10000×. The sequences of all primer pairs used in this study are given in Table S1.

### RNAseq and gene expression analysis

Total RNA was extracted from KSHV and mock infected cells using RNA-Bee (Tel-Test, Inc.). Quality of extracted RNA was verified prior to library preparation on a 2100 Bioanalyzer (Agilent). Strand specific Illumina compatible paired end RNAseq libraries were generated using the ScriptSeq TM v2 RNA-Seq Library Preparation Kit (Epicentre) as per the manufacturer's recommendations. Libraries were sequenced on a HiSeq 2500 system (Illumina). Quality filtered paired end reads were aligned to the human genome (NCBI build 37.2) using the spliced read mapper TopHat2 [139]–[141]. Aligned reads were then assembled and assigned to human transcripts using the Cufflinks2 package [141], [142]. Analysis of differential gene expression, hierarchical clustering of samples and principal component analysis was subsequently performed with the Baggerley's statistical test procedure of the CLC Genomics Workbench v6.5.1 software (Qiagen). For enrichment analysis of functional annotation terms, Gene IDs of genes that were significantly up- or downregulated (p-value < = 0.01, minimal twofold regulation) relative to the mock control were submitted to the DAVID online functional annotation tool (http://david.abcc.ncifcrf.gov/).

## Supporting Information

Figure S1 **ChIP-qPCR of histone modifications at selected loci during** ***de novo*** **infection.** SKL cells were infected with KSHV and chromatin was prepared at indicated time points. Temporal deposition of activating H3K4me3 and repressive H3K27me3 histone marks was evaluated by ChIP-qPCR using specific primers as given in Table S1. Results of ChIP-qPCR experiments are calculated as % of input.
(TIF)

Figure S2 **Co-localization of viral episomes and LANA in SLK cells.** SLK cells were infected with KSHV and analyzed for localization of KSHV episomes and LANA protein at 72 h post infection. Detection of viral episomes was performed by FISH using a KSHV specific probe combined with IF for LANA as described in Protocol S1. Shown are three representative cells (horizontal panels). Images of LANA, Sp100 and the overlay represent maximum intensity projections of z-stacks throughout the nuclei. Colocalization of episomes and LANA was calculated and visualized using Imaris (Bitplane) after 3D reconstruction. Dashed lines indicate the outline of nuclei as determined by DAPI.
(TIF)

Figure S3 **RNA-seq analysis of** ***de novo*** **infected SLK cells.** SLK cells were mock infected (0 h timepoint) or infected with KSHV and harvested after 2, 4, 8, 12, 16, 24, 48 or 96 h of infection. Total RNA was isolated and subjected to RNA-seq analysis as described in materials and methods. (**A**) Graph and table showing the number of up- or downregulated genes (p-value < = 0.01, minimum regulation 2fold) at each time point. See Dataset S2 for individual gene IDs of up- and downregulated genes. (**B**) Hierarchical clustering and (**C**) principal component analysis (PCA) of samples. Clustering, PCA and functional annotation enrichment analysis of up- and downregulated gene (see Datasets S3 and S4, respectively) indicate that gene expression profiles are governed by a cytokine induction phase between 2 h and 4 h post infection, followed by an interferon response that peaks at 12 h of infection and establishment of latent expression patterns after 48 h.
(TIF)

Figure S4 **Transient induction of interferon response genes in KSHV infected SLK cells.** The upper panels show transcript levels of select cytokines and interferon response genes (upper panels) from the RNAseq data given in Dataset S1. The three housekeeping genes GAPDH, ADH5 and VPS29 are shown for comparison (lower panel). Transcript levels are shown as RPKM (reads per kilobase and million mapped reads) values. Baseline expression levels as observed in mock infected cells are marked across plots by a dashed gray line. The fold range (FR) of maximum up- or down-regulation across the entire time course is indicated in each panel. For IFNB1, baseline expression was below the threshold of detection and the maximum upregulation is thus indicated as ‘>100’.
(TIF)

Figure S5 **PML and Sp100 colocalize in KSHV infected cultures.** SLK cells were infected with KSHV for 72 hours (infection rate >95% as established by LANA IF analysis, data not shown) and analyzed by IF staining with antibodies against PML and Sp100. Shown are four representative panels containing 1–2 cells. An overlay of DAPI, PML and Sp100 fluorescence signals is shown in the column labeled ‘overlay’.
(TIF)

Figure S6 **Immunofluorescence staining for PML, Daxx and Sp100 in shRNA expressing EA.hy cells.** EA.hy cells stably expressing shRNAs directed against PML, Daxx or Sp100 were subjected to standard IF-analysis for the individual ND10 components. EA.hy shGFP cells are shown as a control in all panels. Bars indicate distances of 50 µm.
(TIF)

Figure S7 **Depletion of ND10 components facilitates Adenovirus replication in SLK and EA.hy cells.** SLK cells depleted for individual ND10 components (SLK-shDaxx, SLK-shPML and SLK-shSp100) as well as control cells (SLK-shGFP) were infected with wt adenovirus H5pg4100 at a multiplicity of 50 focus-forming units (ffu) per cell. (**A**) Proteins from total-cell extracts were separated by SDS-PAGE and subjected to immunoblotting using 2A6 (α-E1B-55K), B6-8 (α-E2A), and rabbit antiserum to Ad capsid L133. (**B**) Total cell extracts were prepared and treated with proteinase K and PCR was performed using E1B-specific primers (E1B-fw 3′-CGC GGG ATC CAT GGA GCG AAG AAA CCC ATC TGA GC-5′; E1B-rev 3′-CGG TGT CTG GTC ATT AAG CTA AAA-5′). Identical volumes of PCR product were separated on analytic agarose gels (1%) and quantification was performed with the Gene Snap Software (Syngene). (**C**) Viral particles were harvested 48 h post infection and virus yield was determined by quantitative E2A-72K immunofluorescence staining of HEK293 cells. The results represent the averages from three independent experiments and error bars indicate the standard error of the mean. (**D**) EA.hy control cells (EA.hy shGFP) and EA.hy cells depleted for Sp100 (EA.hy shSp100) were infected with wt adenovirus H5*pg*4100 at a multiplicity of 50 ffu per cell. Viral particles were harvested 48 h post infection and virus yield was determined by quantitative E2A-72K immunofluorescence staining of HEK293 cells. The results represent the averages from three independent experiments and error bars indicate the standard error of the mean.
(TIF)

Figure S8 **Immunofluorescence staining for LANA in shRNA expressing EA.hy cells.** EA.hy cells stably expressing shRNAs directed against PML, Daxx or Sp100 or GFP were infected with KSHV for 48 h and subjected to standard IF-analysis for LA\ Mock infected cells are shown as a control in each panel. The number of LANA positive cells was greater than 95% in all cell cultures. Bars indicate distances of 50 µm.
(TIF)

Figure S9 **Immunofluorescence staining for PML and Sp100 in shRNA expressingHUVEC cells.** HUVEC cells expressing shRNAs directed against Sp100 or PML were subjected to standard IF-analysis. HUVEC shGFP cells are shown as a control in all panels. The analysis suggests that individual cells vary with regard to the overall efficiency of the respective knockdown. However, in both cases a substantial fraction of cells exhibits significantly decreased numbers or complete absence of Sp100 or PML-positive nuclear bodies. Bars indicate distances of 50 µm.
(TIF)

Figure S10 **The overall pattern of H3K27me3 is not altered in Sp100 depleted EA.hy cells.** Alternative depiction of the data shown in Figure 10D at the 72 h time point to illustrate that EA.hy-shGFP and EA.hy-shSp100 differ in the degree of H3K27me3 acquisition (note differentially scaled y-axes), but not the overall patterns of acquired H3K27me3 patterns.
(TIF)

Figure S11 **Transcript levels of K2, PAN, ORF73/72/71 and HIF1A in** ***de novo*** **infected SLK cells.** Expression levels of transcripts originating from the viral K2, PAN or major KSHV latency locus (ORF73/72/71), and from the cellular HIF1A gene in mock (0 h) or KSHV infected SLK cells between 2 and 96 h of infection. Transcript levels were analyzed by RNAseq (see complete dataset in Dataset S1) and are indicated as RPKM (Reads per kilobase and million mapped reads) values. Baseline expression levels of HIF1A as observed in mock infected cells are marked by a dashed gray line, and the fold range of expression changes (FR) of HIF1A relative to the mock infected cells is indicated.
(TIF)

Table S1 **Primers used in this study.**
(DOCX)

Dataset S1 **Analysis of gene expression by RNA-seq in mock-infected (0 h time point) and SLK cells that had been infected with KSHV for 2 h, 4 h, 8 h, 12 h, 16 h, 24 h, 48 h or 96 h.** Expression values (RPKM; reads per kilobase and million mapped reads) of individual samples are given in the rightmost columns. Fold changes of expression (relative to mock samples) and corresponding p-values (uncorrected, FDR-corrected, as well as Bonferroni corrected) as determined by Baggerley's test (CLC Genomics Workbench v6.5.1) are given for each time point of infection. The full range of expression values, expression changes and p-values across all time points is given in the leftmost columns.
(XLSX)

Dataset S2 **Up- or downregulated genes in KSHV-infected SLK cells, as used for enrichment analysis of functional annotations.** For each time point after KSHV infection, the dataset lists all Gene IDs from dataset S1 that were considered either up- or downregulated (p-value < = 0.01, minimally two-foldregulation) in infected cells versus the mock control.
(XLSX)

Dataset S3 **Enrichment analysis of functional genes that were upregulated in KSHV-infected cells relative to the mock control.** The output from the online functional annotation tool of the DAVID webservice (http://david.abcc.ncifcrf.gov/) is listed in an individual table for each time point. Shown are all annotations with an enrichment p-value of <0.01. For each annotation, the column “Genes” lists the IDs that were differentially regulated at the time point in question. See the DAVID web site for further details.
(XLSX)

Dataset S4 **Enrichment analysis of functional genes that were downregulated in KSHV-infected cells relative to the mock control.** The output from the online functional annotation tool of the DAVID webservice (http://david.abcc.ncifcrf.gov/) is listed in an individual table for each time point. Shown are all annotations with an enrichment p-value of <0.01. For each annotation, the column “Genes” lists the IDs that were differentially regulated at the time point in question. See the DAVID web site for further details.
(XLSX)

Protocol S1 **Supporting materials and methods.**
(DOCX)
