# Supplementary figures and images for: Influence of ND10 Components on Epigenetic Determinants of Early KSHV Latency Establishment
Source: PLoS Pathog. 2014 Jul 17;10(7):e1004274. doi: 10.1371/journal.ppat.1004274 (PMC4102598; doi:10.1371/journal.ppat.1004274)

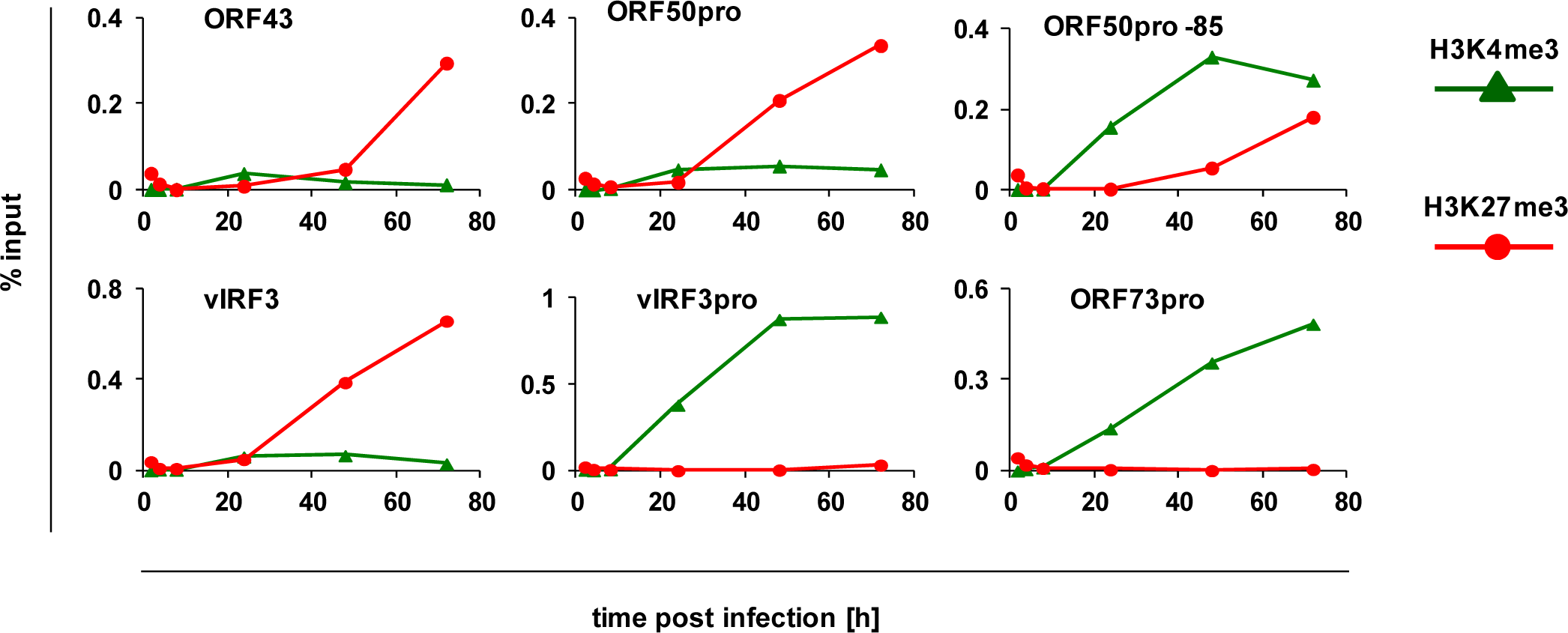

Supplement: Figure S1 — ChIP-qPCR of histone modifications at selected loci during de novo infection. SKL cells were infected with KSHV and chromatin was prepared at indicated time points. Temporal deposition of activating H3K4me3 and repressive H3K27me3 histone marks was evaluated by ChIP-qPCR using specific primers as given in Table S1. Results of ChIP-qPCR experiments are calculated as % of input. (TIF) [file ppat.1004274.s001.tif]

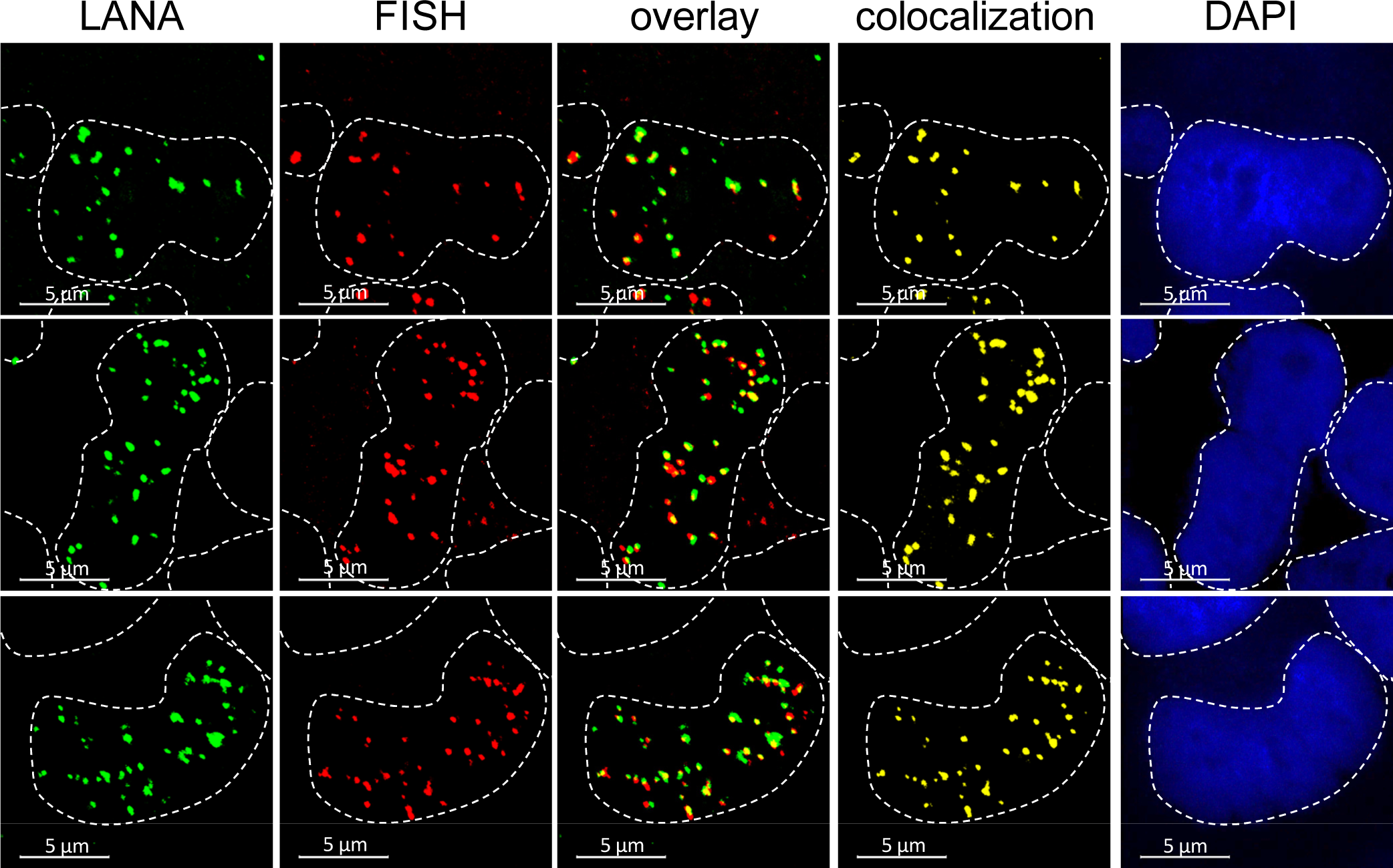

Supplement: Figure S2 — Co-localization of viral episomes and LANA in SLK cells. SLK cells were infected with KSHV and analyzed for localization of KSHV episomes and LANA protein at 72 h post infection. Detection of viral episomes was performed by FISH using a KSHV specific probe combined with IF for LANA as described in Protocol S1. Shown are three representative cells (horizontal panels). Images of LANA, Sp100 and the overlay represent maximum intensity projections of z-stacks throughout the nuclei. Colocalization of episomes and LANA was calculated and visualized using Imaris (Bitplane) after 3D reconstruction. Dashed lines indicate the outline of nuclei as determined by DAPI. (TIF) [file ppat.1004274.s002.tif]

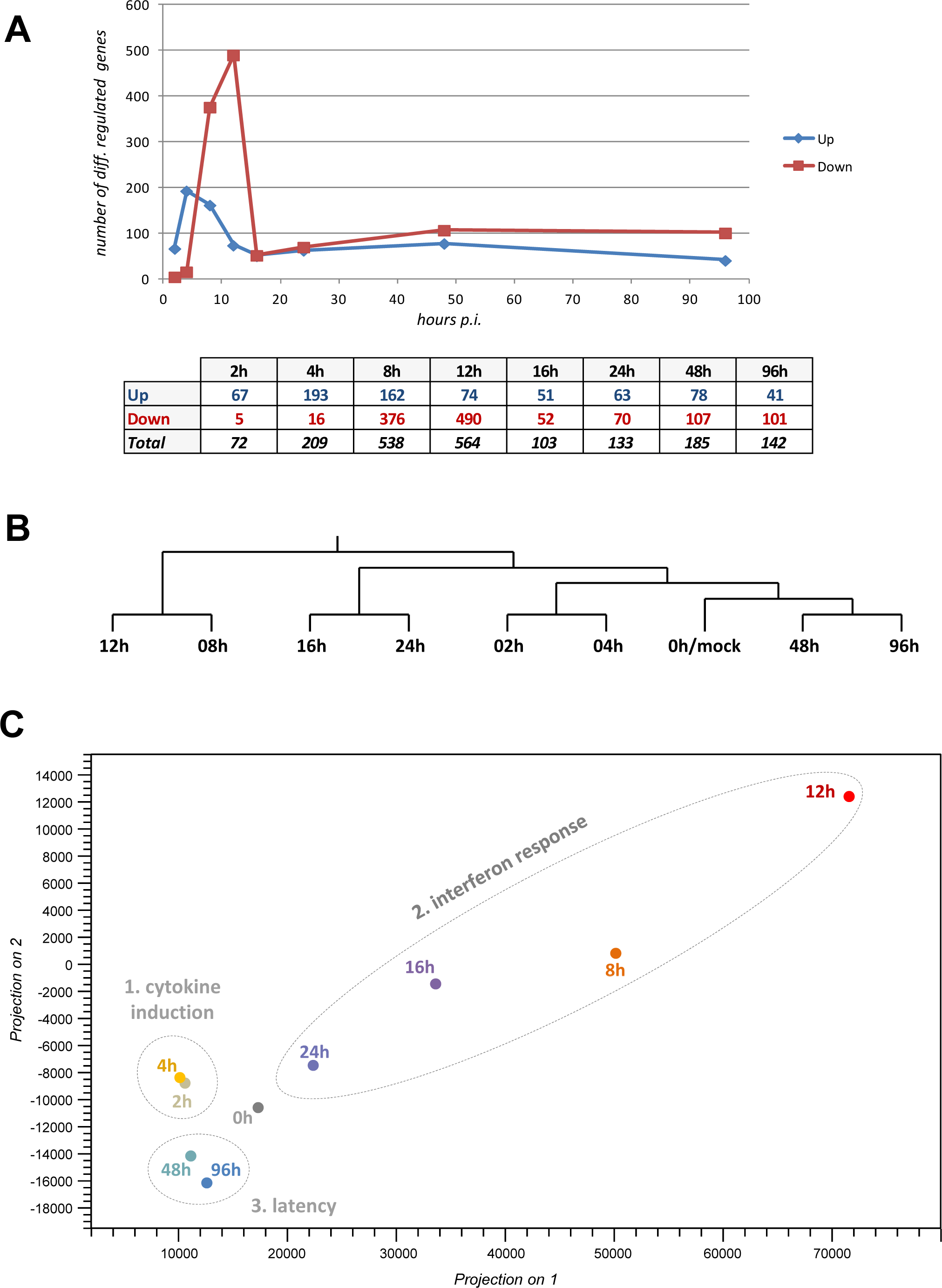

Supplement: Figure S3 — RNA-seq analysis of de novo infected SLK cells. SLK cells were mock infected (0 h timepoint) or infected with KSHV and harvested after 2, 4, 8, 12, 16, 24, 48 or 96 h of infection. Total RNA was isolated and subjected to RNA-seq analysis as described in materials and methods. (A) Graph and table showing the number of up- or downregulated genes (p-value < = 0.01, minimum regulation 2fold) at each time point. See Dataset S2 for individual gene IDs of up- and downregulated genes. (B) Hierarchical clustering and (C) principal component analysis (PCA) of samples. Clustering, PCA and functional annotation enrichment analysis of up- and downregulated gene (see Datasets S3 and S4, respectively) indicate that gene expression profiles are governed by a cytokine induction phase between 2 h and 4 h post infection, followed by an interferon response that peaks at 12 h of infection and establishment of latent expression patterns after 48 h. (TIF) [file ppat.1004274.s003.tif]

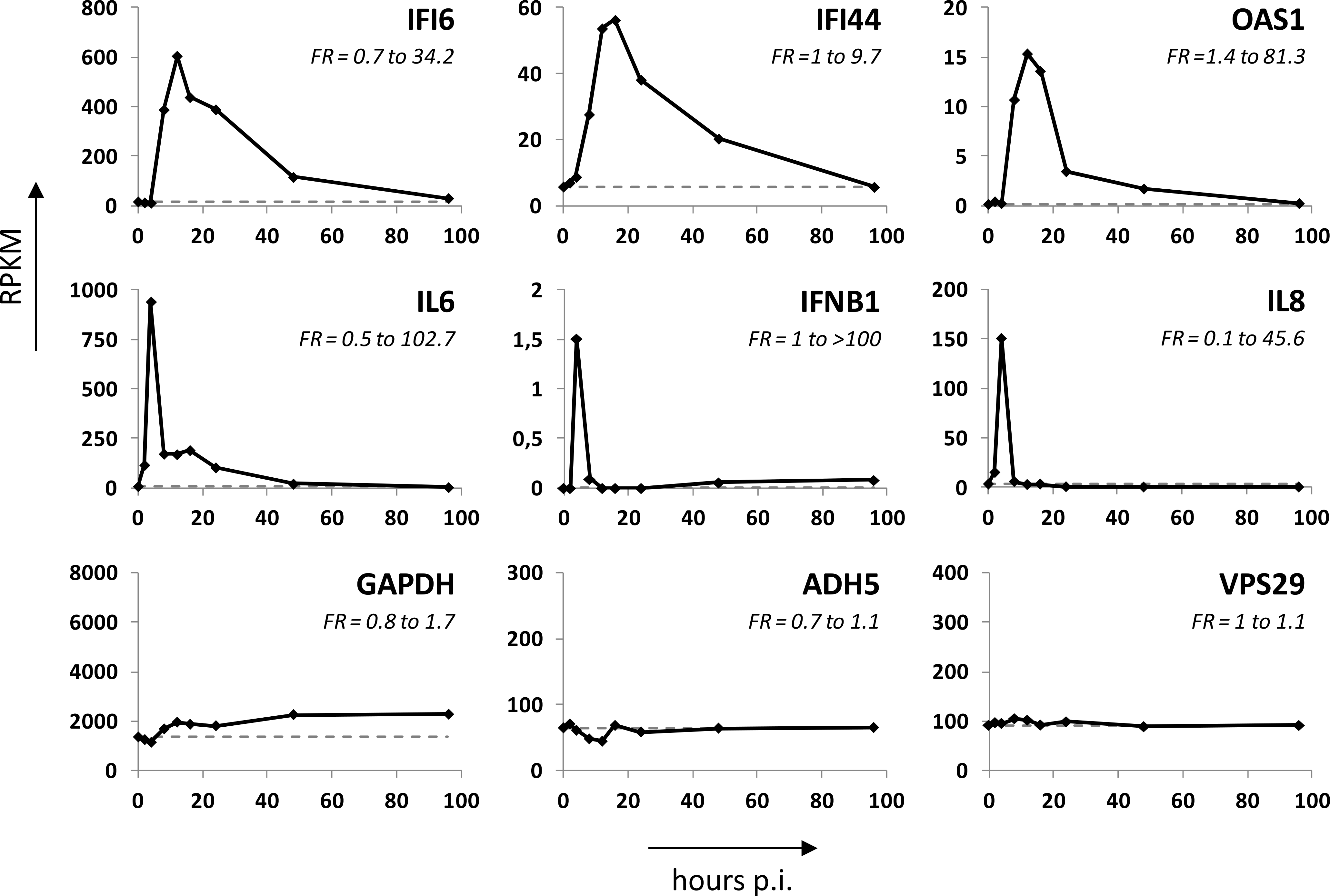

Supplement: Figure S4 — Transient induction of interferon response genes in KSHV infected SLK cells. The upper panels show transcript levels of select cytokines and interferon response genes (upper panels) from the RNAseq data given in Dataset S1. The three housekeeping genes GAPDH, ADH5 and VPS29 are shown for comparison (lower panel). Transcript levels are shown as RPKM (reads per kilobase and million mapped reads) values. Baseline expression levels as observed in mock infected cells are marked across plots by a dashed gray line. The fold range (FR) of maximum up- or down-regulation across the entire time course is indicated in each panel. For IFNB1, baseline expression was below the threshold of detection and the maximum upregulation is thus indicated as ‘>100’. (TIF) [file ppat.1004274.s004.tif]

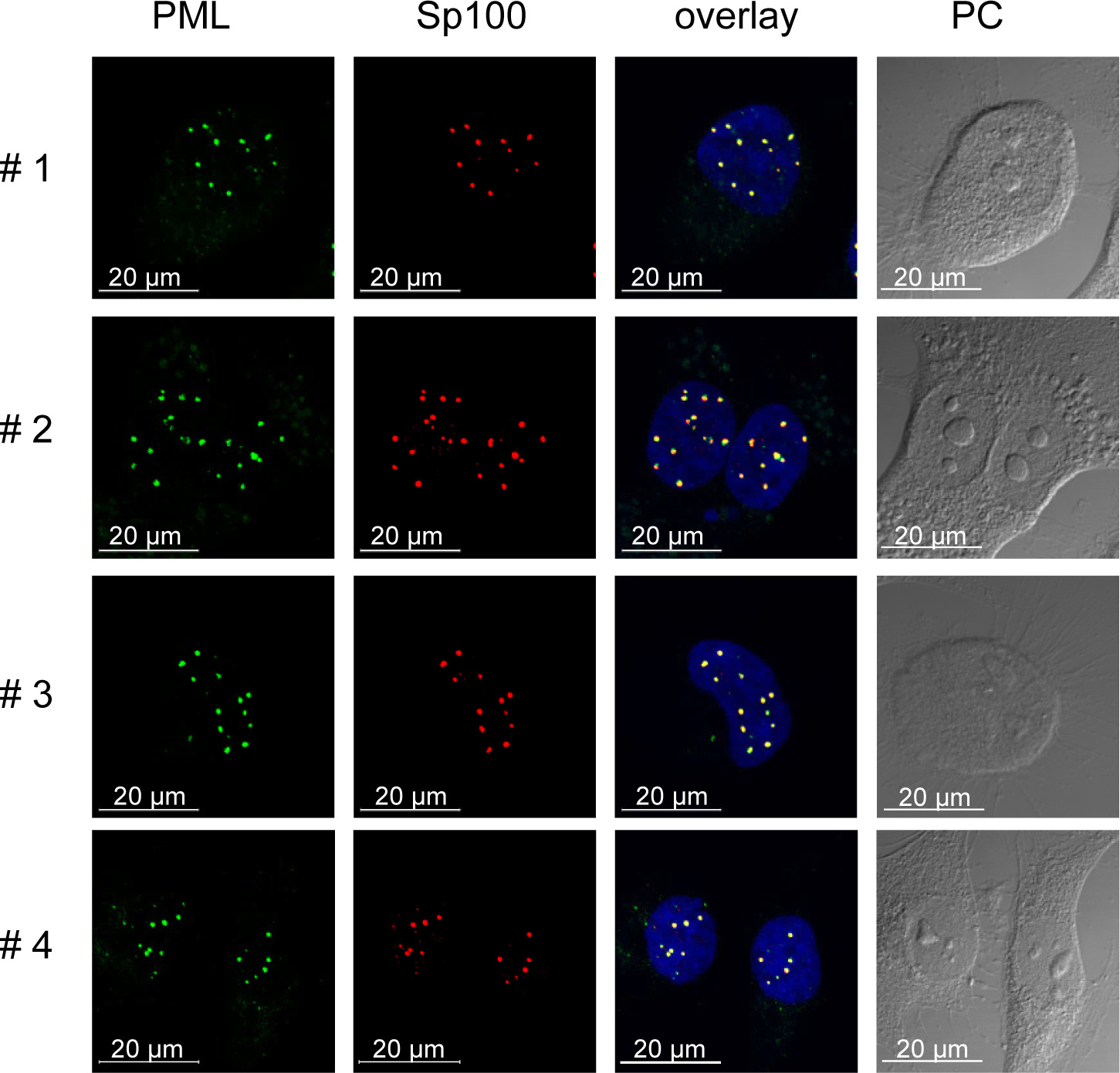

Supplement: Figure S5 — PML and Sp100 colocalize in KSHV infected cultures. SLK cells were infected with KSHV for 72 hours (infection rate >95% as established by LANA IF analysis, data not shown) and analyzed by IF staining with antibodies against PML and Sp100. Shown are four representative panels containing 1–2 cells. An overlay of DAPI, PML and Sp100 fluorescence signals is shown in the column labeled ‘overlay’. (TIF) [file ppat.1004274.s005.tif]

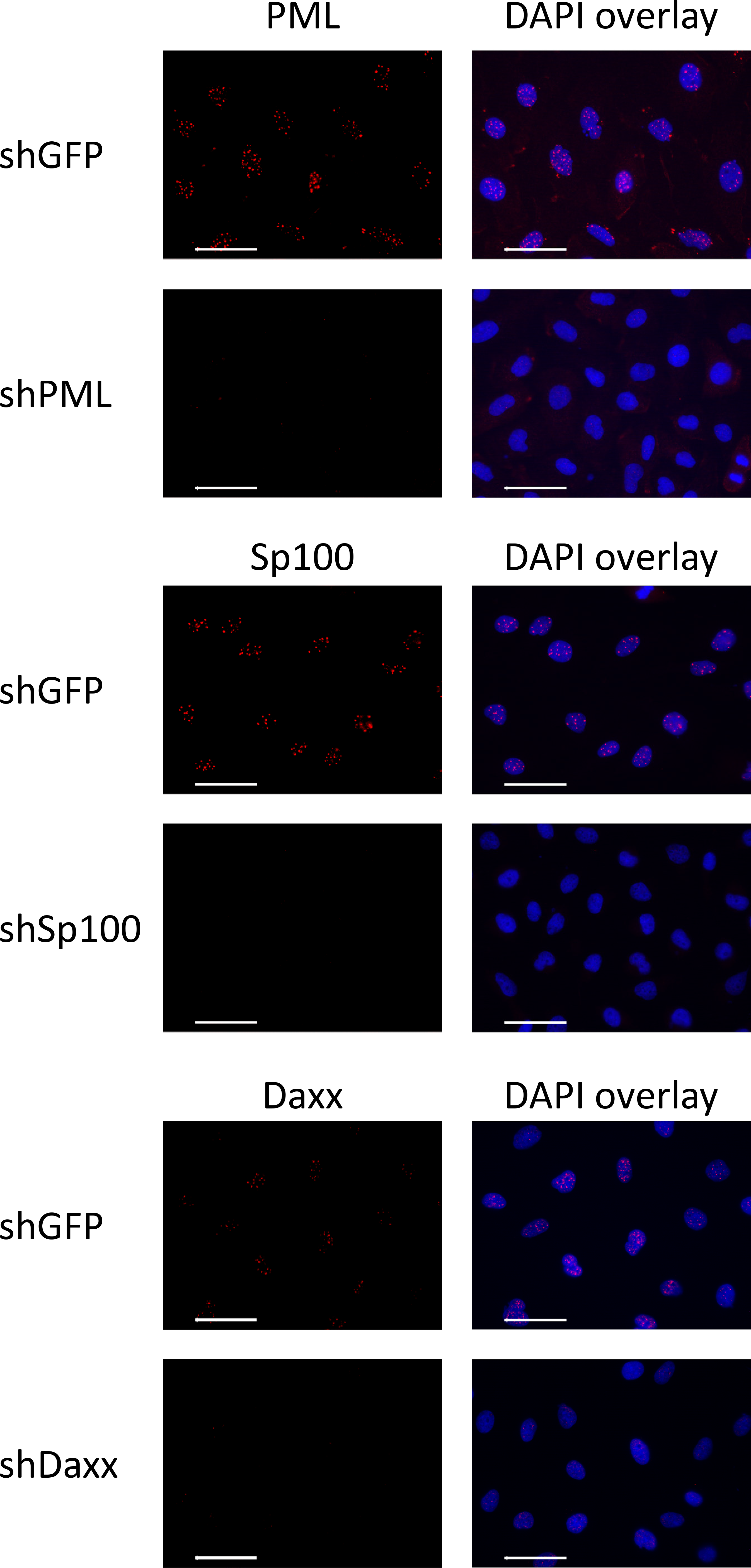

Supplement: Figure S6 — Immunofluorescence staining for PML, Daxx and Sp100 in shRNA expressing EA.hy cells. EA.hy cells stably expressing shRNAs directed against PML, Daxx or Sp100 were subjected to standard IF-analysis for the individual ND10 components. EA.hy shGFP cells are shown as a control in all panels. Bars indicate distances of 50 µm. (TIF) [file ppat.1004274.s006.tif]

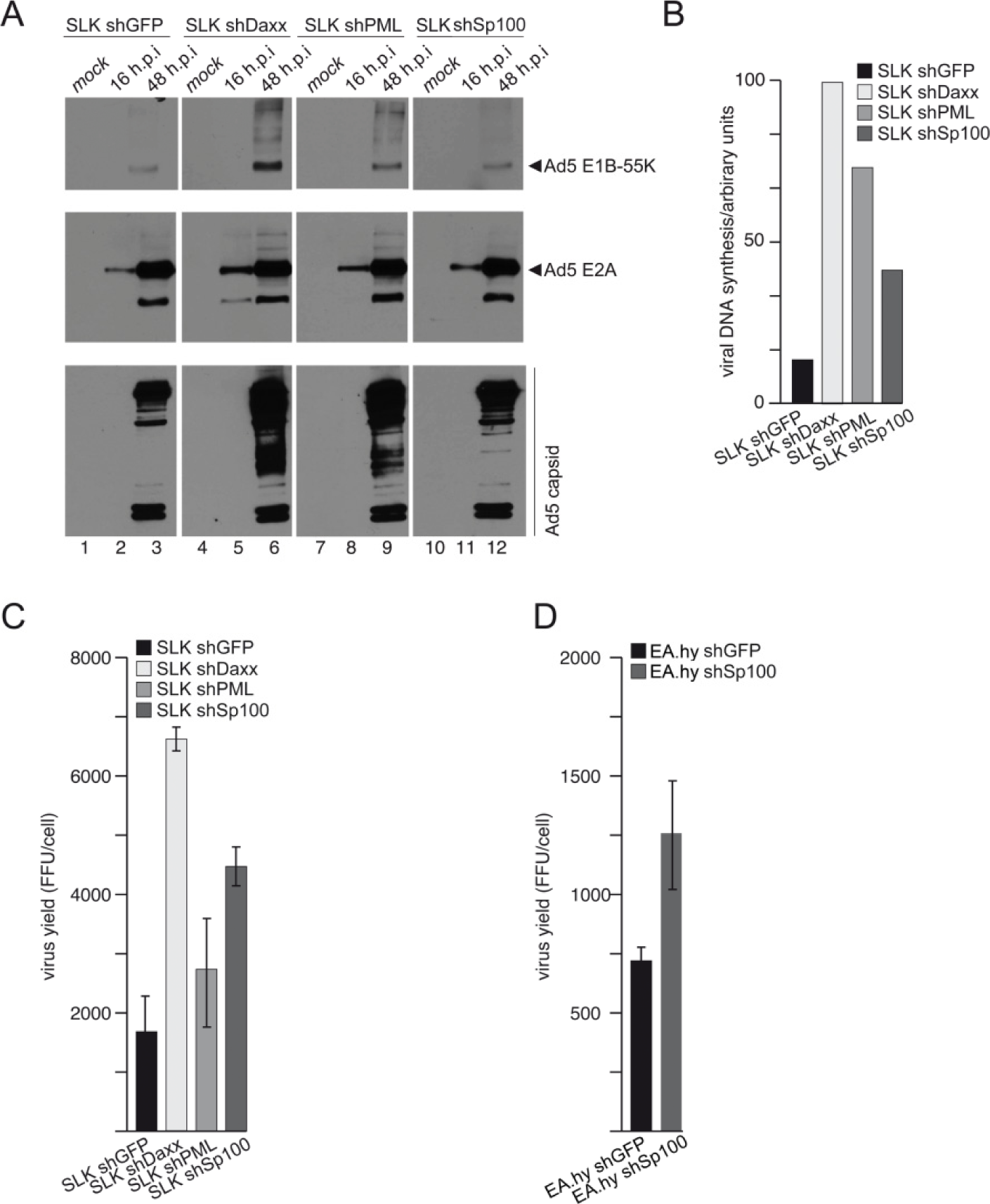

Supplement: Figure S7 — Depletion of ND10 components facilitates Adenovirus replication in SLK and EA.hy cells. SLK cells depleted for individual ND10 components (SLK-shDaxx, SLK-shPML and SLK-shSp100) as well as control cells (SLK-shGFP) were infected with wt adenovirus H5pg4100 at a multiplicity of 50 focus-forming units (ffu) per cell. (A) Proteins from total-cell extracts were separated by SDS-PAGE and subjected to immunoblotting using 2A6 (α-E1B-55K), B6-8 (α-E2A), and rabbit antiserum to Ad capsid L133. (B) Total cell extracts were prepared and treated with proteinase K and PCR was performed using E1B-specific primers (E1B-fw 3′-CGC GGG ATC CAT GGA GCG AAG AAA CCC ATC TGA GC-5′; E1B-rev 3′-CGG TGT CTG GTC ATT AAG CTA AAA-5′). Identical volumes of PCR product were separated on analytic agarose gels (1%) and quantification was performed with the Gene Snap Software (Syngene). (C) Viral particles were harvested 48 h post infection and virus yield was determined by quantitative E2A-72K immunofluorescence staining of HEK293 cells. The results represent the averages from three independent experiments and error bars indicate the standard error of the mean. (D) EA.hy control cells (EA.hy shGFP) and EA.hy cells depleted for Sp100 (EA.hy shSp100) were infected with wt adenovirus H5pg4100 at a multiplicity of 50 ffu per cell. Viral particles were harvested 48 h post infection and virus yield was determined by quantitative E2A-72K immunofluorescence staining of HEK293 cells. The results represent the averages from three independent experiments and error bars indicate the standard error of the mean. (TIF) [file ppat.1004274.s007.tif]

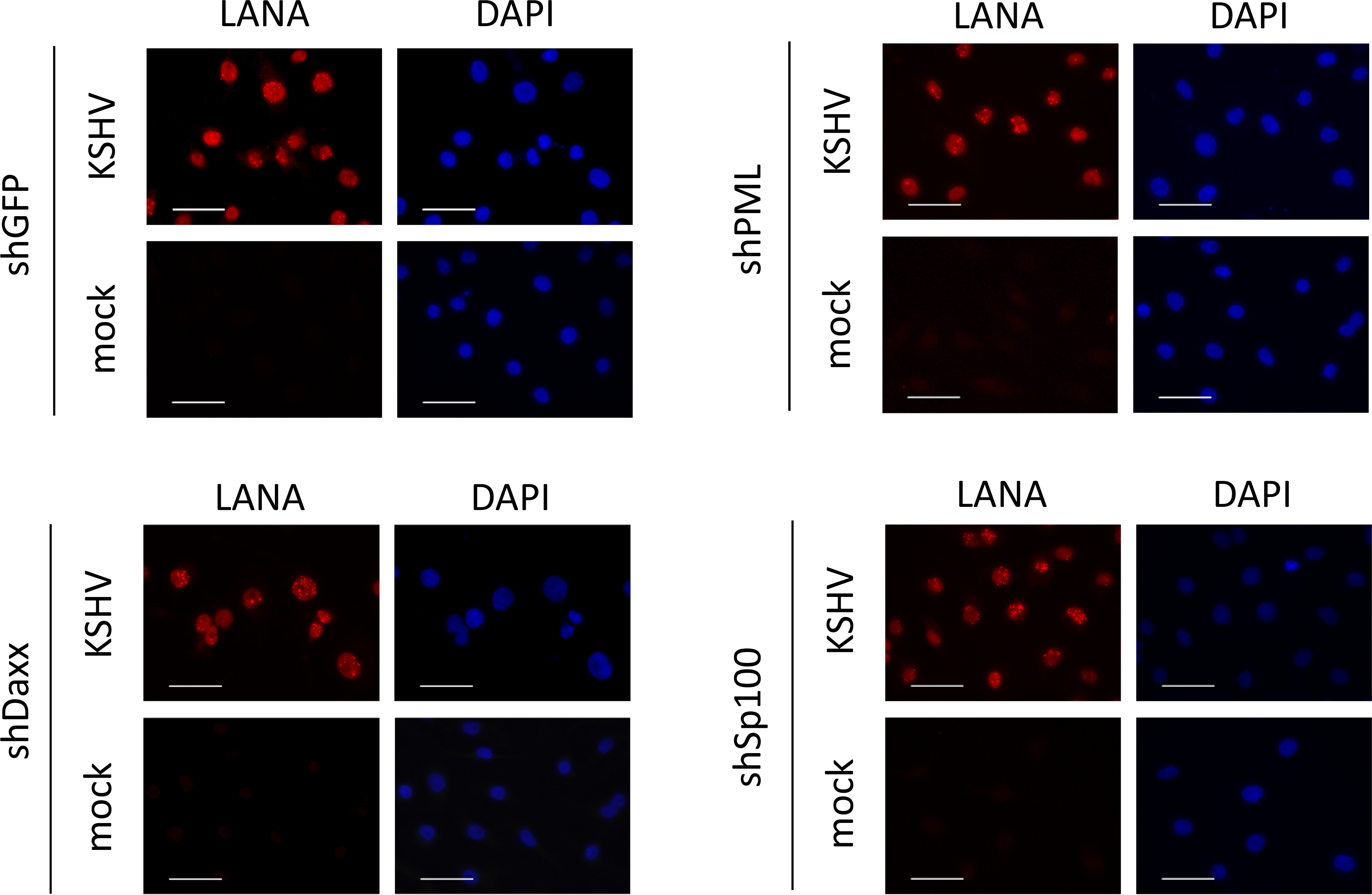

Supplement: Figure S8 — Immunofluorescence staining for LANA in shRNA expressing EA.hy cells. EA.hy cells stably expressing shRNAs directed against PML, Daxx or Sp100 or GFP were infected with KSHV for 48 h and subjected to standard IF-analysis for LA\ Mock infected cells are shown as a control in each panel. The number of LANA positive cells was greater than 95% in all cell cultures. Bars indicate distances of 50 µm. (TIF) [file ppat.1004274.s008.tif]

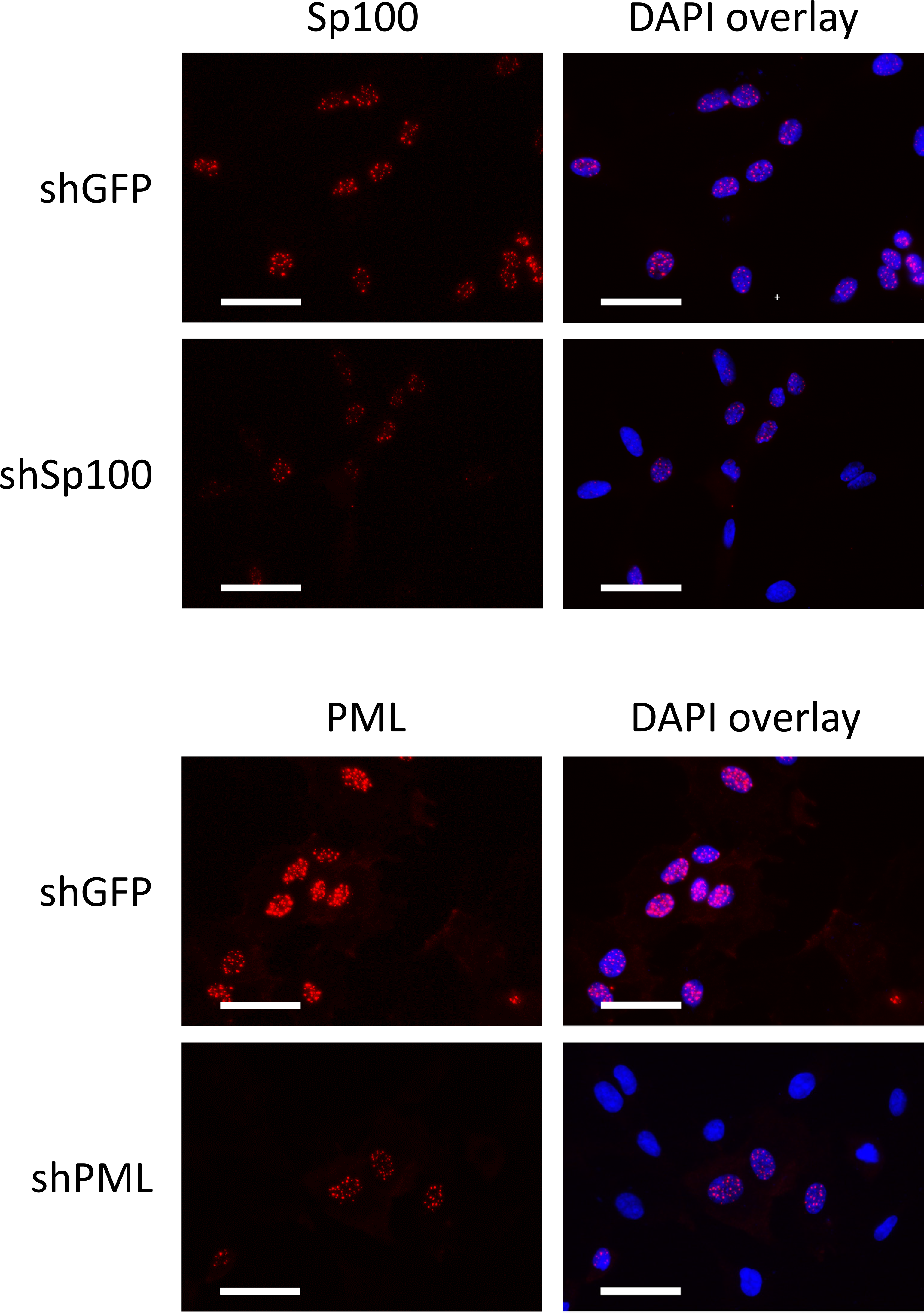

Supplement: Figure S9 — Immunofluorescence staining for PML and Sp100 in shRNA expressingHUVEC cells. HUVEC cells expressing shRNAs directed against Sp100 or PML were subjected to standard IF-analysis. HUVEC shGFP cells are shown as a control in all panels. The analysis suggests that individual cells vary with regard to the overall efficiency of the respective knockdown. However, in both cases a substantial fraction of cells exhibits significantly decreased numbers or complete absence of Sp100 or PML-positive nuclear bodies. Bars indicate distances of 50 µm. (TIF) [file ppat.1004274.s009.tif]

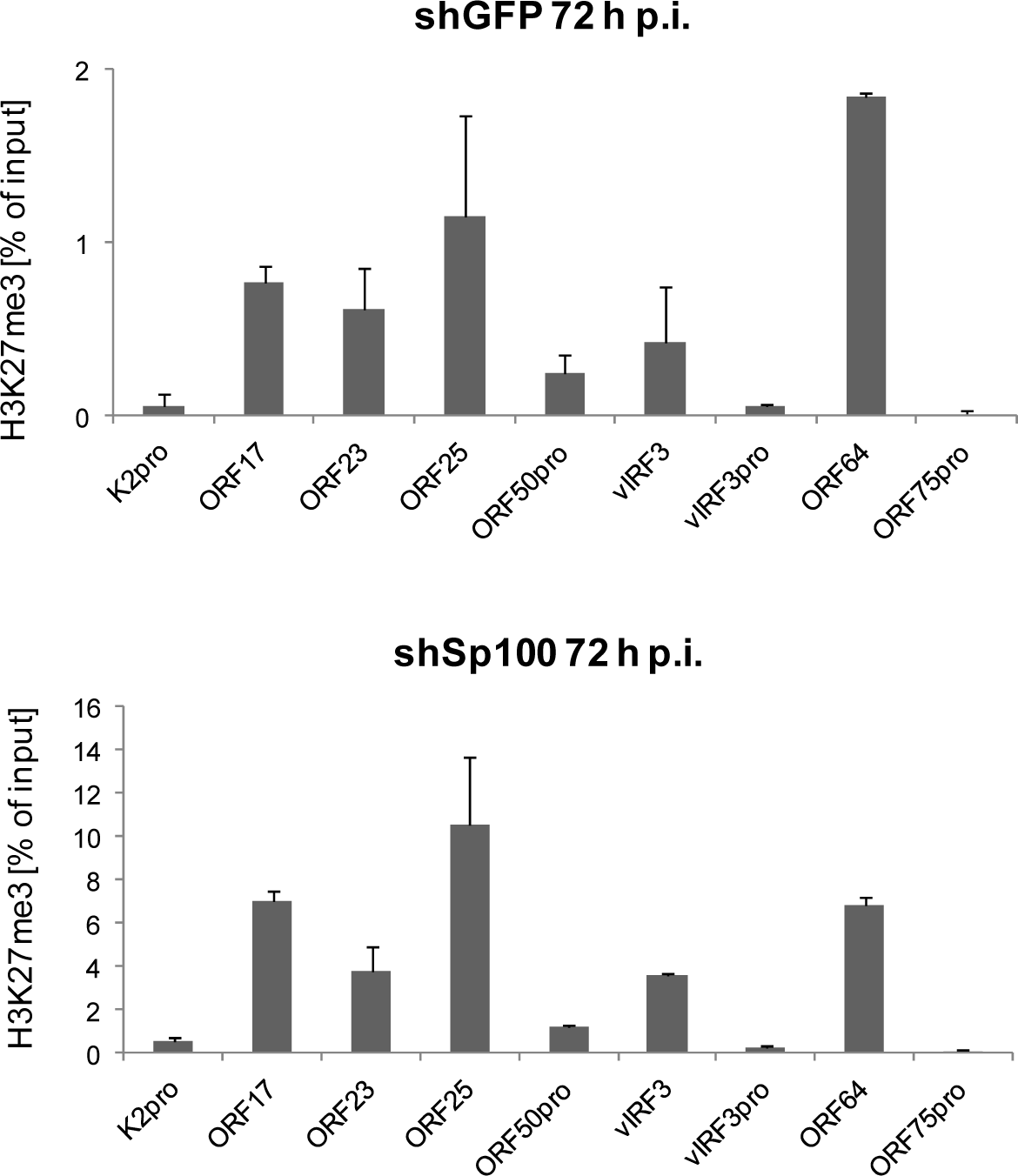

Supplement: Figure S10 — The overall pattern of H3K27me3 is not altered in Sp100 depleted EA.hy cells. Alternative depiction of the data shown in Figure 10D at the 72 h time point to illustrate that EA.hy-shGFP and EA.hy-shSp100 differ in the degree of H3K27me3 acquisition (note differentially scaled y-axes), but not the overall patterns of acquired H3K27me3 patterns. (TIF) [file ppat.1004274.s010.tif]

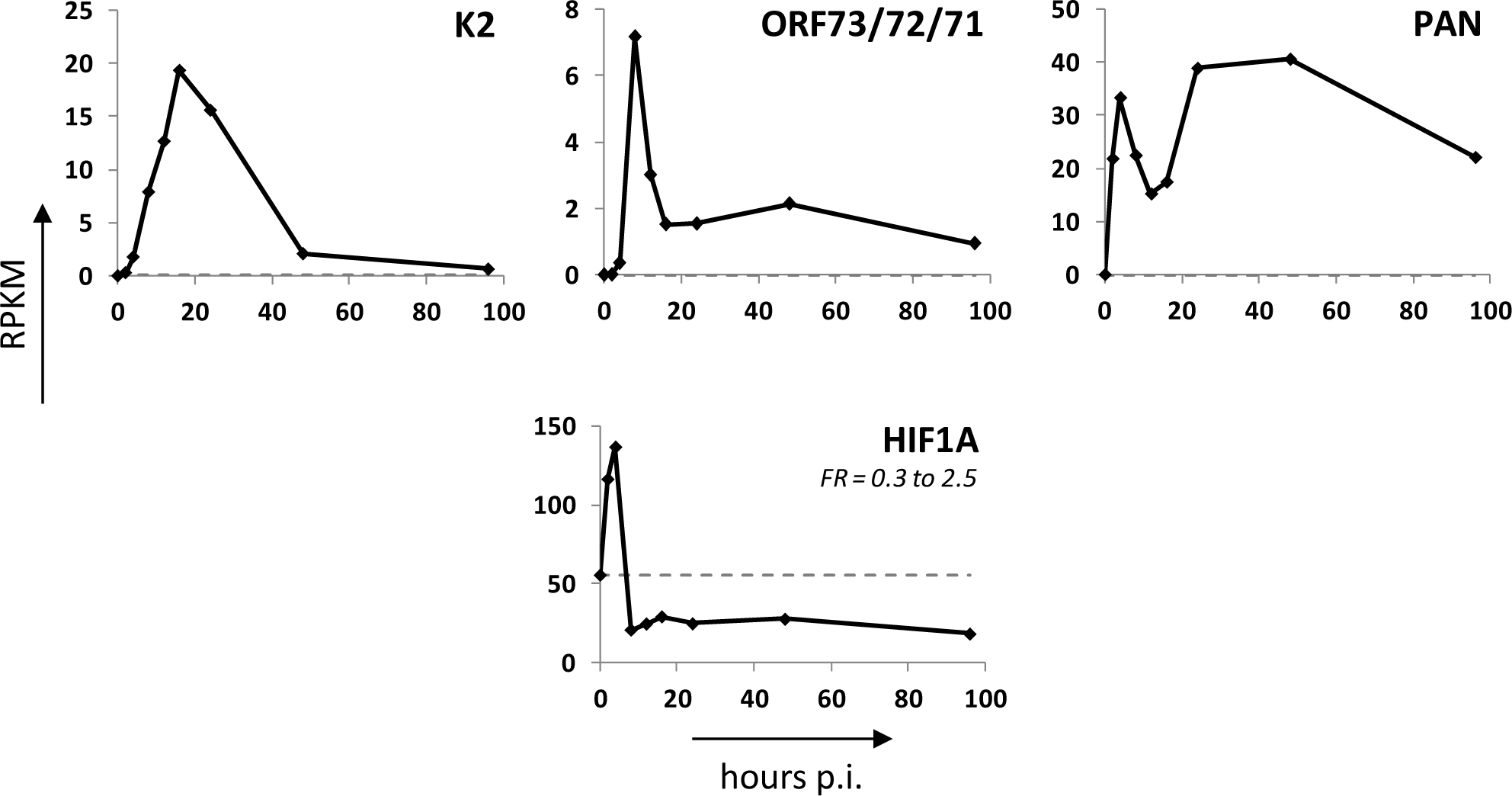

Supplement: Figure S11 — Transcript levels of K2, PAN, ORF73/72/71 and HIF1A in de novo infected SLK cells. Expression levels of transcripts originating from the viral K2, PAN or major KSHV latency locus (ORF73/72/71), and from the cellular HIF1A gene in mock (0 h) or KSHV infected SLK cells between 2 and 96 h of infection. Transcript levels were analyzed by RNAseq (see complete dataset in Dataset S1) and are indicated as RPKM (Reads per kilobase and million mapped reads) values. Baseline expression levels of HIF1A as observed in mock infected cells are marked by a dashed gray line, and the fold range of expression changes (FR) of HIF1A relative to the mock infected cells is indicated. (TIF) [file ppat.1004274.s011.tif]
