# Supplementary material for: Influence of ND10 Components on Epigenetic Determinants of Early KSHV Latency Establishment
Source: PLoS Pathog. 2014 Jul 17;10(7):e1004274. doi: 10.1371/journal.ppat.1004274 (PMC4102598; doi:10.1371/journal.ppat.1004274)
Supplement: Table S1 — Primers used in this study. (DOCX) [file ppat.1004274.s012.docx]

**Primers used for qPCR analyses.**

| Primer | Sequence |
| --- | --- |
| ORF43fw | CTACCGTGACCACCCAGTCT |
| ORF43rv | CTGCTTCTCAATGCCATCAA |
| ORF50profw | TCCGAGGTAATGTGCTCTATGAAG |
| ORF50prorv | ACAGACACCGGAGCAATACCC |
| ORF50pro-85fw | TACCGGCGACTCATTAAG |
| ORF50pro-85rv | TTGCGGAGTAAGGTTGAC |
| vIRF3fw | AAAAATTCGCCAACAACTGG |
| vIRF3rv | CCAGAATGTAGCAGGGGAAT |
| vIRF3profw | GCGGTAAGACAAAGGGAGGT |
| vIRF3prorv | TACCTTGCCCCATTTTACCA |
| ORF73profw | CCCGTGCTGACATAGTTAGCG |
| ORF73prorv | GGTACTGGGTCTGAACCACCAC |
| K2profw | GCGTTCCAGATACCAGCAGT |
| K2prorv | TAGTGTATGCCGCGTTAGCA |
| K5profw | GTTCCCCACCTCTTCCCTAC |
| K5prorv | CTCCCCTTTCCCTTTTTCAG |
| ORF17fw | gtggctgagcaaaaaggatg |
| ORF17rv | tgtcaattcccgacaatgaa |
| ORF23fw | ACACGACACGATGTTTTCCA |
| ORF23rv | TCATGGAGCGTGCTAACAAC |
| ORF25fw | ggtccaccccttctttgatt |
| ORF25rv | gcgagcggttgtggtatatt |
| ORF62fw | TGGTCACGAAGGTACTGTGG |
| ORF62rv | CTCATGGACACTGGGGAGTT |
| ORF64fw | CAGGCCGTATCAATTCCAGT |
| ORF64rv | TCGGAGGATACCAGGTTTTG |
| ORF75profw | AGCGAGCACCGTCTGTATTT |
| ORF75prorv | GCACCGGAGGCTACTATCTG |
| TermRepsfw | TGTGTGTGAGCCTGTTTG |
| TermRepsrv | TGTTCACGTAGTGTCCAG |
| ORF50mRNAfw | GAGAATACTGTCCAGGCAGCCAC |
| ORF50mRNArv | AGTTGGGCCTTCAGTTCGTCC |
| ORF59mRNAfw | TTCGTTGGAGTGCCAAATCAGC |
| ORF59mRNArv | CGGGTTAGCCTGGAGTCCTTAATC |
| ORF73mRNAfw | tgggtgagtgtggaggtgta |
| ORF73mRNArv | ccaccgctttcaagtcctac |
| GAPDHfw | AACAGCGACACCCACTCCTC |
| GAPDHrv | CATACCAGGAAATGAGCTTGACAA |
