# Supplementary material for: Influence of ND10 Components on Epigenetic Determinants of Early KSHV Latency Establishment
Source: PLoS Pathog. 2014 Jul 17;10(7):e1004274. doi: 10.1371/journal.ppat.1004274 (PMC4102598; doi:10.1371/journal.ppat.1004274)
Supplement: Protocol S1 — Supporting materials and methods. (DOCX) [file ppat.1004274.s017.docx]

**Supporting Methods**

***Adenovirus infection and evaluation of virus growth***

H5*pg*4100 served as the wild type Ad5 parental virus in these studies [[1](#_ENREF_1)]. Viruses were propagated, titrated and infected as described previously [[2](#_ENREF_2)]. Virus yield was determined by quantitative E2A-72K immunofluorescence staining and viral DNA replication was monitored by quantitative PCR exactly as described previously [[3](#_ENREF_3)]. Primary Ab specific for Ad proteins used in this study included E1B-55K mab 2A6 [[4](#_ENREF_4)], E2A-72K mouse mab B6-8 [[5](#_ENREF_5)] and anti-capsid rabbit polyclonal antibody [[2](#_ENREF_2)].

***Fluorescence in situ hybridization (FISH)***

*Sample preparation:* For FISH analysis 5 x 10^4^ were centrifuged on Superfrost/Plus slides (Fisher) for 5 min at 900 rpm and fixed in methanol. Cells were treated with 0.01 % pepsin / 0.01 N HCl for 5 min followed by RNA digestion (100 µg / ml RNAseA in 2 x SSC: 0.3 M NaCl, 0.03 M sodium citrate) at 37 °C for 1 h at 37 °C. Cells were washed with PBS and fixed again in 3 % formaldehyde (in PBS with 50 mM MgCl2). Samples were dehydrated by incubation in increasing concentrations of ethanol (70 %, 85 %, and 100 %) for 5 min each. Samples were air dried. Samples were denatured by incubation in 70 % formamide (in 2 x SSC for 5 min at 73 °C) and immediately placed in ice cold 70 % ethanol for 5 min. Dehydration was repeated as described above.

*Design of a KSHV detection probe:* KSHV-cosmid SChyB23 (1µg), which contains approx. 36.000 bp of the KSHV genome (ORF59 – ORF75), was labeled using Nick Translation Mix (Roche, 11745816910) according to the manufacturer’s instructions. Labeled probe DNA was precipitated with ethanol in the presence of sonicated salmon sperm DNA (Life Technologies), which served as a carrier. Precipitated DNA was collected in hybridization buffer (50% formamide, 10% dextran sulfate, 2 x SSC).

*Probe hybridization and immunofluorescence analysis:* The DNA probe (50 ng) was denatured for 5 min at 73°C and placed under a coverslip covering the respective sample prepared as described above. The coverslip was fixed using fixogum (Marabu, 290110000). Hybridization was performed in a humid chamber overnight at 37°C. After hybridization, slides were washed three times alternating at 20 °C at 70°C and again at 20°C (2 x SSC, 0.2% Tween) for 2 min each, followed by blocking with 4 % BSA in PBS for 30 min at 37°C. Detection of the probe and LANA was performed with a cy3-labeled anti biotin antibody (1:50) and a LANA specific antibody (1:500) in 4 % BSA / PBS, 0.2% Tween), for 2h at 37°C. Primary LANA antibody was detected with an Alexa-488 labeled anti rabbit antibody as described under IF analysis. Slides were then washed three times (PBS, 0.2 % Tween) for 10 min at 20°C, and mounted with Vectashield mounting Medium with DAPI (Vector, H-1200). To analyze colocalization of LANA and episomes, 3D-reconstructions and the colocalization channel were generated using Imaris software (Bitplane) and standard settings.

***References***

1. Groitl, P. and T. Dobner, *Construction of adenovirus type 5 early region 1 and 4 virus mutants.* Methods Mol Med, 2007. **130**: p. 29-39.

2. Kindsmuller, K., et al., *A 49-kilodalton isoform of the adenovirus type 5 early region 1B 55-kilodalton protein is sufficient to support virus replication.* J Virol, 2009. **83**(18): p. 9045-56.

3. Schreiner, S., et al., *Control of human adenovirus type 5 gene expression by cellular Daxx/ATRX chromatin-associated complexes.* Nucleic Acids Res, 2013. **41**(6): p. 3532-50.

4. Sarnow, P., C.A. Sullivan, and A.J. Levine, *A monoclonal antibody detecting the adenovirus type 5-E1b-58Kd tumor antigen: characterization of the E1b-58Kd tumor antigen in adenovirus-infected and -transformed cells.* Virology, 1982. **120**(2): p. 510-7.

5. Reich, N.C., et al., *Monoclonal antibodies which recognize native and denatured forms of the adenovirus DNA-binding protein.* Virology, 1983. **128**(2): p. 480-4.
